# Supplementary material for: Climate change, biodiversity loss, and Indigenous Peoples’ health and wellbeing: A systematic umbrella review
Source: PLOS Glob Public Health. 2024 Mar 20;4(3):e0002995. doi: 10.1371/journal.pgph.0002995 (PMC10954122; doi:10.1371/journal.pgph.0002995)
Supplement: S1 Table — (DOCX) [file pgph.0002995.s002.docx]

S1 Table. Excerpt of quality appraisal data chart.

| **Citation** | **9. Degree of attentiveness to/recognition of colonialism as an antecedent to and driver of the climate-health pathways being explored, as well as its historic and ongoing impact on Indigenous Peoples' health and wellbeing** | **10. Level of Indigenous Peoples' involvement in the research (e.g., co-design, identification of research question(s), contribution of Indigenous knowledges, perspectives, or values to interpretation of findings)** | **11. Extent to which the record discusses the relevance of the findings to Indigenous Peoples' priorities and processes** | **12. Extent to which the record centres Indigenous-led strategies and responses, and focuses on advancing Indigenous health and wellbeing** |
| --- | --- | --- | --- | --- |
| Schlingmann et al. (2021). Global patterns of adaptation to climate change by Indigenous Peoples and local communities. A systematic review. Current opinion in environmental sustainability, 51, 55-64. | Low  Colonialism not explicitly discussed. | Low  No information provided as to the level of Indigenous Peoples' involvement. | Medium  Record is focused on Indigenous Peoples and Local Communities, so not solely focused on IP. Nevertheless, the main aim of the research is to examine localized responses to climate change impacts, and in that regard, is very relevant to IP's priorities and processes. | Low  Though the article is not explicitly focused on IP, rather more broadly the adaptation processes among "Indigenous and local communities". |
| Ford et al. (2012). Mapping human dimensions of climate change research in the Canadian Arctic. Ambio, 41(8), 808-822. | Low  Colonialism not explicitly discussed. | High  p.810- review initiated by/developed in collaboration with federal knowledge users; ITK; Nunavut Research Institute; researchers | Low  Focus is on the state of knowledge/scholarship and direction of research and conceptual contribution, rather than directly connected to the Indigenous Peoples' priorities and processes with respect to adaptation, response to climate-health risks, etc. | Low  Article is not explicitly focused on health (wellbeing, by considering societal dimensions of climate change broadly, and many sectors implicated). But as a result, not explicitly focused on advancing Indigenous well-being, (though implied). |
| Gupta et al. (2022). Community-based responses for tackling environmental and socio-economic change and impacts in mountain social–ecological systems. Ambio, 51(5), 1123-1142. | Low  Colonialism not recognized because of global/eco-regional focus and the diversity of community histories included. | Low  No IP involvement in this review reported. | Medium  Not explicit to Indigenous Peoples (though they are the majority in the mountain SES being discussed); lots of emphasis on engagement of communities; local-level decision-making; etc. (pgs.1136-37). Not high however because the record discusses relevance of findings to regional areas which includes Indigenous Peoples, but does not explicitly discuss Indigenous relevance or priorities | Low  Not explicitly focused on Indigenous Peoples. Recommendations are very focused on engagement with/empowerment of local communities; not explicitly health or wellbeing focused, but implied based on discussion of social/ecological changes. |
| Markkula et al. (2019). A review of climate change impacts on the ecosystem services in the Saami Homeland in Finland. Science of the Total Environment, 692, 1070-1085. | Low  No mention of colonialism within this regional and cultural context. | Medium  The survey was designed in collaboration with Metsähallitus (The Finnish Forest Administration), Reindeer Herders' Association (RHA), Finnish Environment Institute (SYKE), and Universities of Lapland and Jyväskylä. | Low  The record does incorporate results of the survey of Saami reindeer herders but does not extend these perspectives beyond including them in the review summaries. | Low  These ideas are touched upon, particularly in the discussion of the concept of traditional ecological knowledge, but they are not further developed into specific strategies, responses or recommendations. |
| Borish et al. (2022). Relationships between Rangifer and Indigenous Well-being in the North American Arctic and Subarctic: A Review Based on the Academic Published Literature. Arctic, 75(1), 86-104. | Low  However, the article is not focused explicitly on impacts of climate change/biodiversity loss on Indigenous wellbeing, rather characterizing relationships between a (declining) species and Indigenous Peoples. Not as intuitive/relevant to make connection to colonialism, given the framing/focus. | High  This systematic review process was informed by a Caribou Research Steering Committee situated in Labrador, Canada, with both Indigenous and non-Indigenous members spanning a range of disciplinary expertise, sectors, and knowledge systems (13 members in total) | High  Focused on developing future research/learning "what we need to know" about Rangifer-related changes, in order to support mental/emotional wellbeing; community adaptation; intergenerational knowledge sharing with youth about Rangifer. | High  Supports/explicitly names the continued need for collaborative partnerships between researchers, Indigenous Peoples, governments, organizations; inclusion of multiple forms of knowledge/science; supporting Indigenous Peoples' wellbeing in relation to the changes they are and will continue to experience with respect to Rangifer species (caribou/reindeer). |
| Charnley et al. (2022). Drought-related cholera outbreaks in Africa and the implications for climate change: a narrative review. Pathogens and global health, 116(1), 3-12. | N/A  Included as per weighted criteria, therefore article is not specific to Indigenous Peoples | N/A  Included as per weighted criteria, therefore article is not specific to Indigenous Peoples | N/A  Included as per weighted criteria, therefore article is not specific to Indigenous Peoples | N/A  Included as per weighted criteria, therefore article is not specific to Indigenous Peoples |
| Pearce et al. (2011). Advancing adaptation planning for climate change in the Inuvialuit Settlement Region (ISR): a review and critique. Regional Environmental Change, 11, 1-17. | Low  Little attention given to colonialism or other antecedents/systemic drivers to climate-health pathways and impacts on Indigenous health and wellbeing | Medium  No evidence or explicit statement of Indigenous involvement in the review process; however, there existed an in-field component that involved visiting the ISR and gathering literature available in regional and community libraries and organization records so perhaps more involvement in actuality. | Medium  The record discusses priorities in response to the vulnerability framework used to guide the analysis. | Low  Indigenous values or centring Indigenous-led strategies are absent, despite focus on advancing Indigenous health and wellbeing. |
| Davis et al. (2022). Shifting safeties and mobilities on the land in Arctic North America: a systematic approach to identifying the root causes of disaster. *Sustainability*, *14*(12), 7061. | High  pg.2 - extensive discussion of colonialism and colonial policy in relation to mobilities and environmental/climatic change (in the introduction). | Low  Not explicitly reported. | High  pg.13-14 (discussion), e.g., recognizing the need to "[do] justice to local agency, while attending to the external structures that create risk" and the challenges of this. Platforming localised Indigenous-led action, along with institutions that create structural violence and risk. | High  pg.13-14- explicit discussion of "positive drivers of access to land"/strengths-based approach; community-driven processes of creativity and innovation. |
| Ford et al. (2012). Research on the human dimensions of climate change in Nunavut, Nunavik, and Nunatsiavut: a literature review and gap analysis. Arctic, 289-304. | Low  No explicit discussion of colonialism as an antecedent to climate-health impacts being discussed; implicit in the broader sociocultural changes being discussed in Inuit society, but not named as being rooted in colonialism. | High  p.290 - knowledge users involved in the research team; also, a very collaborative approach, as "the study was commissioned by knowledge users who were concerned that duplication of research was contributing to research fatigue in communities and needed to know where to prioritize future efforts. All team members were actively engaged in the project, from research design to analysis and interpretation of results" | High  Not necessarily focused on self-determination in climate-health research (despite lots of discussion about needed research/gaps). | Medium  Focus on advancing Inuit health/wellbeing by encouraging community-based adaptation/monitoring/surveillance of climate-health impacts; acknowledgement of adaptation and community-based efforts (strengths-based lens). Could be strengthened to be more explicitly supportive of advancing Inuit health and wellbeing. |
| Lam et al. (2019). Community-based monitoring of Indigenous food security in a changing climate: global trends and future directions. *Environmental Research Letters*, *14*(7), 073002. | High  Analysis and reporting supports critical inquiry that attends to historic imbalance of power, attention, and collaboration with Indigenous peoples in research. While colonialism and imperialism are not discussed extensively, they are acknowledged as antecedents to these dynamics in footnotes. | Low  The level of IP involvement in the review is not evident. Yet, the authors pay particular attention to the degree to which the records included in their review report/consider IP contributions and perspectives. | Medium  Findings are discussed in terms of relevance to general IP priorities regarding power/control/inclusion within research, but are not directed at community/population-directed priorities specific to this review. | Medium  The record aims to assess the level of Indigenous leadership or involvement is reported and pays attention to this. Also considers how Indigenous knowledges fit into CBM. The record does explicitly centre Indigenous-led strategies or responses, however, despite focusing ultimately on methods to advance Indigenous wellbeing. |
| Ford et al. (2010). Vulnerability of Aboriginal health systems in Canada to climate change. Global Environmental Change, 20(4), 668-680. | High  Specific sub-section of results focused on socio-political inequality and the broader context in which Indigenous Peoples' rights and ability to act independently are constrained by colonial influences. Recognition of colonialism and Indigenous relation to the State made very clear, alongside implications from these dynamics related to climate impacts/adaptation ability. | Low  No Indigenous involvement reported in this study. | High  Record discusses needs for Indigenous rights and sovereignties to be upheld, as well as calls for future actions to be in partnership with IPs and guided by their priorities/identified needs. p.677 - advocates for the need for "more effective partnerships with Aboriginal communities and organizations and culturally relevant knowledge translation" | Medium  Record focused on advancing Indigenous people's wellbeing, but does not report a strengths-based approach inclusive of Indigenous values. Focused on advancing wellbeing vis-a-vis strengthening Indigenous health systems to adapt/respond to climate-change-induced stressors. |
| Loring & Gerlach (2015). Searching for progress on food security in the North American North: a research synthesis and meta-analysis of the peer-reviewed literature. *Arctic*, 380-392. | High  Authors use critical inquiry throughout paper to acknowledge systemic antecedents/drivers of pathways identified and their impact on Indigenous health and wellbeing. | Low  No Indigenous involvement was reported in the review process. | Medium  Record discusses relevance of findings to the priorities of Indigenous peoples insomuch as advancing a rights-based discourse addresses this criteria. Specific Indigenous-defined priorities and processes are absent from the record. | Medium  The record does not centre Indigenous-led strategies and responses, but does advocate for them as well as focuses on advancing the health and wellbeing of Indigenous Peoples. |
| Ford (2012). Indigenous health and climate change. American journal of public health, 102(7), 1260-1266. | High  Colonialism explicitly discussed as well as other aspects such as power imbalances, prominence of Eurocentric knowledge ways being used in projects with Indigenous Peoples is. Barriers to and history/current experiences of oppression through medical system also acknowledged. p.1262 - discussion of colonial history/ongoing reality; challenges of land rights/sovereignty, assimilationist policies, etc. that create enhanced sensitivities/exposures to climate-change risks for Indigenous Peoples. | Low  No Indigenous involvement reported in the review process. | Medium  Focused on the broader structural/socioeconomic determinants of Indigenous health and well-being, the underlying root causes of vulnerability/sensitivity to climate risks. Discussion of community empowerment; no explicit discussion of self-determination in research, etc. | High  Recognition of Indigenous conceptualizations of health and wellbeing; the need to strengthen adaptive capacity and support Indigenous communities' adaptation efforts. Record frames the robust opportunities that traditional knowledge systems hold for appropriate adaptation strategies that support Indigenous health and wellbeing. |
| King & Furgal (2014). Is hunting still healthy? Understanding the interrelationships between indigenous participation in land-based practices and human-environmental health. International Journal of Environmental Research and Public Health, 11(6), 5751-5782. | Medium  pg. 5760: "in the reviewed literature, the importance of intergenerational knowledge transfer about how to "catch" was commonly approached as a loss of connection to land through colonisation and dispossession, and the consequences this had for all members of communities to engage in traditional land-based practices. Indeed, the adverse and ongoing impacts of colonisation featured strongly in many papers reviewed. The tension between traditional and contemporary, Indigenous experiences and aspirations were commonly filtered through the pervasive lens of the legacy of colonisation." | Low  No Indigenous involvement reported in the review process. | Low  Focus was more on providing evidence through more holistic research and policy approaches, rather than practical application. | Low  No Indigenous-led strategies profiled, beyond challenging biomedical constructs of health. |
| Dannenberg et al. (2019). Managed retreat as a strategy for climate change adaptation in small communities: public health implications. Climatic change, 153, 1-14. | Low  Not discussed - Indigenous Peoples not explicitly the focus (even though the majority of the community members in these small communities are Indigenous) | Low  Indigenous Peoples not explicitly the focus (even though the majority of the community members in these small communities are Indigenous) | Low  Indigenous Peoples not explicitly the focus (even though the majority of the community members in these small communities are Indigenous) | Medium  Indigenous Peoples not explicitly the focus (even though the majority of the community members in these small communities are Indigenous). In that light, the focus on advancing the health/wellbeing of these communities extends to that of Indigenous Peoples. |
| Kipp et al. (2019). The need for community-led, integrated and innovative monitoring programmes when responding to the health impacts of climate change. International journal of circumpolar health, 78(2), 1517581. | Low  No mention of colonialism or antecedent drivers explored in a critical sense. | Low  No involvement of Indigenous Peoples explicitly mentioned. Workshop discussed, but unclear the level of participation of Indigenous Peoples. | Medium  Record discusses relevance of findings to Indigenous needs in the circumpolar North, but in as much as experts and literature have discussed. Not in terms of specific directives given by community/IP for this research. | Medium  Reporting advances call for more Indigenous-led research and draws attention to the gaps noted in their review, despite Indigenous involvement. focused on community based monitoring and the creation of locally and culturally useful technological innovation. |
| Jaakkola et al. (2018). The holistic effects of climate change on the culture, well-being, and health of the Saami, the only indigenous people in the European Union. Current environmental health reports, 5, 401-417. | Medium  Societal changes and assimilation policies noted generally in conclusions related to loss of language and cultural knowledge. Recognition of historical influences of State hegemony (not colonialism for this context) as critical inquiry, and the challenges this poses to Saami. | Low  Within 'Compliance with Ethical Standards' it is noted that the summary of the main findings will be made available for the Saami communities in North Saami language, however it is not indicated whether or not communities were consulted at any stage of the review. | Low  Findings are relevant to Saami priorities in as much as the ethnographic field study that was included informed the authors, which is unclear. | Medium  Indigenous-led strategies not centred, but record does focus on advancing Indigenous health and wellbeing. Also incorporates Saami understandings of lifestyle to frame analysis. |
| Jasmine et al. (2016). Traditional knowledge systems in India for biodiversity conservation. | Low | Low | Low | Low |
| Hillier et al. (2021). Examining the concept of One Health for indigenous communities: A systematic review. One Health, 12, 100248. | Medium  pg.1 - up-front in the framing of the introduction is an acknowledgement of colonialism as the root of health inequity. The discussion mentioned the role that colonialism and climate change play in disrupting Indigenous pathways to health and wellness, but not incorporated explicitly in analysis. | Low  No Indigenous involvement reported in the review process. | Medium  Throughout article, focused on aligning the One Health concept with Indigenous conceptualizations of health/wellbeing and ways of doing and being. Record discusses the potential alignments of One Health approaches to health interventions as aligning with Indigenous knowledges and prioritizing Indigenous priorities. Nothing mentioned beyond this, though. | Medium  pg.5 - "There is an urgent need to envision a distinctive yet collaborative and respectful One Health approach that happens alongside with Indigenous Peoples and organizations". Indigenous values and cultural understandings were occasionally highlighted from the primary research reported on. The record does focus on advancing Indigenous wellbeing, but no Indigenous-led strategies were reported. |
| Eerkes-Medrano & Huntington. (2021). Untold Stories: Indigenous Knowledge Beyond the Changing Arctic Cryosphere. Frontiers in Climate, 3, 675805. | High  Not explicit discussion of colonialism, but other discussions of national sovereignty; broader political/social changes that affect Arctic Indigenous Peoples' lives, beyond climate-change related impacts; supports critical inquiry. | Low  Not directly involved in this research, but reference throughout the article to Indigenous Peoples' perspectives shared with the researchers over years (e.g., researchers have been presumably embedded in the context and integrated their learnings/perspectives into the current study). | High  Article centres the voices/"untold stories"/experiences of Arctic Indigenous Peoples and the other priorities/concerns they have, beyond climate change-induced, cryosphere-related changes. | High  Focused on examining health impacts, and impacts to Arctic Indigenous societies more broadly, from the changing cryosphere, with an emphasis on how research efforts can build a "more complete, comprehensive story" around these impacts. Strengths-based approach, underscoring peoples' adaptation and responses; Indigenous perspectives on the changes, and hope. |
| Little et al. (2021). Drivers and health implications of the dietary transition among Inuit in the Canadian Arctic: a scoping review. Public health nutrition, 24(9), 2650-2668. | High  Attentiveness to colonialism as antecedent and driver of climate-health pathways clear, and ultimately supportive of critical inquiry. | Medium  Acknowledgements section identifies Inuit organization partners reviewed the manuscript and offered their contributions to it. The level of contribution is not specified. | High  Findings are relevant to Indigenous people's priorities, especially as acknowledgements indicate Inuit input on the manuscript and its priority/processes framing. | Medium  Strengths-based approach inclusive of Indigenous values does not appear evident, but the manuscript is focused on advancing Indigenous health and wellbeing. |
| McNamara et al. (2021). Exploring climate-driven non-economic loss and damage in the Pacific Islands. Current Opinion in Environmental Sustainability, 50, 1-11. | Low  No information provided on these structural determinants within this region. | Low  No details provided on Indigenous Peoples' involvement. | N/A  Included as per weighted criteria, therefore article is not specific to Indigenous Peoples | Medium  There is awareness indicated of a strengths-based approach to community and the power of social cohesion within Indigenous communities, but the article could go further in terms of focusing on advancing Indigenous health and well-being (was also included as per our weighted criteria) |
| Jones et al. (2020). Climate change mitigation policies and co-impacts on indigenous health: A scoping review. International Journal of Environmental Research and Public Health, 17(23), 9063. | High  Records pays particular attention to colonialism and history/ongoing inequitable impacts on Indigenous health & wellbeing | High  Research led by two Indigenous scholars | High  Record discusses relevance of findings to Indigenous priorities and processes well | High  Authors overall conclusion is how the inclusion and further addition of approaches led by and inclusive of Indigenous values, which is essential for advancing IP wellbeing. |
| Bryson et al. (2020). Neglected tropical diseases in the context of climate change in East Africa: a systematic scoping review. The American journal of tropical medicine and hygiene, 102(6), 1443. | Low  Focus not on Indigenous people's health, but excerpt discussing IP's particular sensitivities to climate change did not include recognition of colonialism. | N/A  The article was not explicitly focused on Indigenous Peoples, however, and so this would not have been expected. | N/A  The article was not explicitly focused on Indigenous Peoples, however, and so this would not have been expected. | Low  Review is not focused on Indigenous Peoples; however, still a mention of the importance of further research as to the inequitable impacts of climate change on Indigenous Peoples' health in East Africa, in relation to NTDs. |
| Vogliano et al. (2021). Progress towards SDG 2: Zero hunger in melanesia–A state of data scoping review. Global Food Security, 29, 100519. | Medium  Colonization and globalization may have increased the vulnerability of most Pacific Island states by weakening traditional social structures and undermining traditional management practices through the introduction of mono-cultured agricultural practices and imported processed food items. | Low  One author is affiliated with the Solomon Islands National University, but it is not clear their positionality or contributions in designing or conducting the review. | Medium  There are several instances throughout the paper where reference is made to ensuring Pacific Islander values are at the heard of planning for sustainable and effective interventions; Common themes from successful nutrition education strategies to reduce NCD risk among Indigenous peoples include a dedicated focus on the Indigenous population, widespread community involvement and integration of local health workers, and a focus on high risk individuals | Medium  Reference is made throughout to traditional or Indigenous knowledge and the importance of leveraging this knowledge to improve food security and sovereignty and therefore health and wellbeing within this region. |
| Ingemann et al (2020). Patient experience studies in the circumpolar region: a scoping review. BMJ open, 10(10), e042973. | Medium  Colonialism and medical history acknoweldged as important factor in health systems serving Indigenous peoples, but not in relation to climate-health pathways or ongoing imp"act on IP health; "The predominant approaches to health system evaluation are rooted in a biomedical conception of health, though other approaches, such as postcolonial and traditional knowledge ideologies are beginning to become incorporated into mainstream health systems analysis." | Low  No Indigenous involvement reported in review process. | Low  Record is not focused on Indigenous peoples nor their priorities/processes, although does mention that lived experience and patient-identified priorities are worthwhile in future research. | Low  Does not centre Indigenous led strategies or responses, but does acknowledge lessons should be learned from records that report these things in future efforts/research. |
| Middleton et al. (2020). Indigenous mental health in a changing climate: a systematic scoping review of the global literature. Environmental Research Letters, 15(5), 053001. | High  Critical inquiry is used throughout the record to guide/ interpret the work. Colonialism and other drivers of climate-health pathways were explored as having ongoing impacts on Indigenous health and wellbeing. | High  "Ongoing engagement and collaboration with Indigenous research partners and leaders guided the conceptualization, analyses, and interpretation of findings for this review." | High  Record discusses relevance of findings to Indigenous priorities for future research requiring IP involvement. | Medium  Analysis uses strengths-based approach centering Indigenous-led strategies/responses as much as data allows, but Indigenous values not used to centre the research. However, focus of record is on advancing IP wellbeing. |
| van Bavel et al. (2020). Contributions of scale: what we stand to gain from Indigenous and local inclusion in climate and health monitoring and surveillance systems. Environmental Research Letters, 15(8), 083008. | Low  Article discussed history but not explicitly linked to colonialism. | Low  The focus of the review was to examine multiple and diverse knowledge systems in climate-health surveillance, however there was no mention of any involvement of Indigenous research partners in the complex design of this review. | Low  Findings indicate that neither scientific, Indigenous nor local knowledge systems alone will be able to contribute to the breadth and depth of information necessary to detect and inform action along pathways of climate-health impact. | Medium  The literature search aimed to include locally inclusive or participatory approaches along with multiple and diverse knowledge systems: By continuing to reference and explain local and Indigenous processes using the same methodologies and concepts taken from Western science, not only do we lose meaning, but we also delegitimize other ways of knowing, and even jeopardizing the opportunities of being able to work together; researchers, scientists, local and Indigenous communities. |
| Akearok et al. (2019). Identifying and achieving consensus on health-related indicators of climate change in Nunavut. Arctic, 72(3), 289-299. | Low  Though perhaps would not have made as much sense to acknowledge the driving colonial influence behind climate change/health impacts - as focused on developing a set of place-specific indicators (e.g., a slightly different type of article). | High  Research is embedded in Nunavut; involved a collaborative/consensus-building workshop with Nunavummiut; oriented by an Inuit community research model; results based on Nunavummiut perspectives on indicators. | High  Article discusses the importance of community-based/led monitoring; community-led approaches to determining priorities for study and intervention to address mitigation/adaptation (p.297). | High  Article is focused on Nunavut Inuit perspectives on what indicators are place-specific; culturally-relevant; implicitly contributing to advancing Inuit wellbeing and supporting Inuit-led efforts to mitigate/adapt to climate-health impacts in the Arctic. |
| Sahu et al. (2022). Measuring Impact of Climate Change on Indigenous Health in the Background of Multiple Disadvantages: A Scoping Review for Equitable Public Health Policy Formulation. Journal of Prevention, 1-36. | High  Critical inquiry is included in the situating and interpretation of the findings. | Low  No involvement of IP reported in this research. | High  Record discusses relevance of findings to Indigenous peoples broadly and advances priorities and processes related to Indigenous control, rights, leadership, etc. | Medium  The record does not centre Indigenous-led strategies, but does advance the call for more research/approaches inclusive of Indigenous values and being focused on IP health and wellbeing. |
| Kiddle et al. (2021). An Oceania urban design agenda linking ecosystem services, nature-based solutions, traditional ecological knowledge and wellbeing. Sustainability, 13(22), 12660. | Low  A number of Indigenous concepts were noted in this review, however, the historical processes and related on-going impacts of urbanization within this region are not recognized specifically. | Unsure (unclear)  In the second last sentence there is indication of on-going research undertaken by a collaboration of researchers and practitioners from this region, including the authors. | Medium  Climate change adaptation is disscussed as needing to be informed by TEK and relevant to those living within Oceania, specifically (so directly tied to the priorities and processes of Indigenous Peoples within Oceania). | High  Focused on advancing IP wellbeing: "need, potential‚ and therefore opportunity‚ to define an urban design agenda positioned within an urban ecosystem services framework, focused on human wellbeing and informed by traditional ecological knowledge, determined by and relevant for those living in the islands of Oceania as a means to work towards effective urban climate change adaptation." |
| Reis et al. (2022). Specific Environmental Health Concerns and Medical Challenges in Arctic and Sub-Arctic Regions. Health, (3), 22. | Low  Not mentioned at all (though the article is not focused explicitly on IP). | Low  No involvement of IP reported in this research. | Low  Findings of more relevance to countries within the region, rather than Indigenous groups. | Low  Indigenous-led strategies and responses are not described (though also not explicitly focused on IP). |
| Cottrell (2022). Avoiding a new era in biopiracy: Including indigenous and local knowledge in nature-based solutions to climate change. Environmental Science & Policy, 135, 162-168. | Medium  Colonialism was not discussed, but the legacy of the scientific community appropriating and/or extracting from IPLCs without compensatory action was made. In other words, power imbalances and the importance of Indigenous agency were attended to. | Low  No explicit involvement in the research. | Medium  Record promotes the need for Indigenous sovereignty and partnership in NbS projects/approaches in global sense, but does not mention localized or diversity of priorities/processes. Yet p.167 - discussion about prioritizing Indigenous sovereignty; importance of IPLC involvement in NbS projects, and doing so in adherence with particular principles/values. | Medium  Focuses on advancing Indigenous wellbeing vis-a-vis supporting more inclusion of IPLCs in NbS projects, and doing so in accordance with particular values. Record promotes the need for Indigenous sovereignty and partnership in NbS projects/approaches. |
| Zimmermann et al. (2023). A leverage points perspective on Arctic Indigenous food systems research: a systematic review. Sustainability Science, 1-20. | High  There is growing recognition that transformative change is needed to resolve the challenges Indigenous communities across the Arctic face today. Deep-rooted colonial influences are at the center of many of these issues, and there is recognition that Indigenous communities need to exercise sovereignty over their food systems to improve Indigenous health and well-being and adaptability to current and future challenges. | Medium  It is acknowledged that co-created research processes can support decolonization and initiate actions relevant to Indigenous communities to leverage their ability to influence transformational change. | Medium  Actively engaging Indigenous voices throughout research processes will facilitate the recognition of multiple knowledge systems and inform scientific practices to help them become sensitive to the needs and desires of marginalized groups and non-human actants. Following the principles of knowledge co-production can help to systematically reflect on power positions and sources of inequity. | Medium  The need for plurality in understanding sustainability transformations through the active engagement of Indigenous Knowledge holders in research processes has recently been formulated. We extend this call for research on Arctic Indigenous food systems and encourage future scholars to reflect on their research approaches, strive for a plurality of knowledge and actively contribute to the decolonization of research practices. |
| Lebel et al. (2022). Climate change and Indigenous mental health in the Circumpolar North: A systematic review to inform clinical practice. Transcultural Psychiatry, 59(3), 312-336. | High  Critical inquiry is recognized and attended to in analysis, reporting, and discussion of findings. | Low  No Indigenous involvement reported in the review. | Medium  Record discusses relevance of findings to IP priorities and processes in as much as the extensive qualitative research enables. Yet, this review was not informed by a particular group's priorities and processes, especially given the authors' note of the unequal representation of Circumpolar Peoples in this review (mostly Inuit within Canada). | High  Record centres strengths-based adaptation responses where found in the review and focuses on advancing Indigenous health and wellbeing. Lacking inclusion of Indigenous values, however. |
| Shafiee et al. (2022). Food Security Status of Indigenous Peoples in Canada According to the 4 Pillars of Food Security: A Scoping Review. Advances in Nutrition, 13(6), 2537-2558. | Low  A sentence on pg 17 states, "As Indigenous peoples move away from consuming traditional foods to market foods as a result of disrupted traditional food systems due to colonization, several factors may drive Indigenous peoples' food choices towards low-quality and nutrient-poor market foods; The authors suggested that changing food preferences and knowledge, poverty and socioeconomic factors, climate change, and colonial processes are drivers of this transition (but fairly limited engagement with colonialism beyond this). | Low  None indicated, although the work was supported by the Network Environments for Indigenous Health Research (NEIHR). | Low  Majority of recommendations are geared towards future research to address gaps revealed. | Medium  Recommendations specific to policy change states the importance of decolonizing food and knowledge systems. |
| Hagen et al. (2022). Climate change-related risks and adaptation potential in Central and South America during the 21st century. Environmental Research Letters, 17(3), 033002. | Low  Though the article is not explicitly focused on Indigenous Peoples, rather is scoped geographically/regionally. | Low  Though the article isn't explicitly focused on Indigenous Peoples. | Low  Emphasis on supporting Indigenous communities' involvement/leadership in adaptation/management; yet findings are region-wide. Considerations are made for minority/vulnerable populations in which Indigenous Poeples are included. | Medium  Throughout results (adaptation measures sections), authors discuss the need for Indigenous-led management; inclusion of Indigenous knowledge; improved communications with communities as to risks, etc. Focus is not explicitly on Indigenous health/wellbeing, however. |
| Leal Filho et al. (2022). Understanding responses to climate-related water scarcity in Africa. Science of the Total Environment, 806, 150420. | Low  No critical inquiry used to recognize antecedent forces of climate-health pathways and impact on IP. | Low  No mention of IP involvement in this review. | Unsure (unclear)  Relevance of findings at regional scale, not specific to Indigenous peoples. However, findings acknowledge the importance of local/autonomous responses to climate risks. | Medium  Record highlights one or two Indigenous-led strategies and responses, but does not centre them nor acknowledge Indigenous values or advancing Indigenous wellbeing. |
