# Supplementary material for: Climate change, biodiversity loss, and Indigenous Peoples’ health and wellbeing: A systematic umbrella review
Source: PLOS Glob Public Health. 2024 Mar 20;4(3):e0002995. doi: 10.1371/journal.pgph.0002995 (PMC10954122; doi:10.1371/journal.pgph.0002995)
Supplement: S2 Table — (DOCX) [file pgph.0002995.s003.docx]

**S2 Table. Summary of studies and extracted data pertaining to key findings, observations, and recommendations.**

| **Citation** | **Key Findings/Conclusions about the Relationships Studied** | **Any Further Comments/Observations/**  **Relevant Data** | **Recommended Strategies to Address Impacts** |
| --- | --- | --- | --- |
| Schlingmann et al. (2021). Global patterns of adaptation to climate change by Indigenous Peoples and local communities. A systematic review. Current opinion in environmental sustainability, 51, 55-64. | Results on global patterns of local responses to climate change impacts show that ILK is relevant and transferable beyond the local context and scale of communities. While the similarities in response strategies across climates may seem surprising, we argue that the patterns reflect the fact that people use similar strategies, rather than identical responses. | Main focus is on responses to face climate change impacts. | For future work, the authors recommend: the consideration of additional literature, including grey literature, could further improve the classification system and understandings of local responses to climate change. Future research could also apply this classification system to related topics such as assessments of adaptation drivers, adaptation, enablers, and barriers. |
| Ford et al. (2012). Mapping human dimensions of climate change research in the Canadian Arctic. Ambio, 41(8), 808-822. | The review highlights that while current scholarship develops a baseline understanding of the HDCC in the eastern and central Canadian Arctic, key gaps are evident.  "Traditional Knowledge is Widely Recognized as Essential for Documenting and Understanding the HDCC" (p.814)  Results indicate high prevalence of geographic disparities in research. Majority of reviewed work focuses on subsistence hunting, fishing, trapping, and emphasizes vulnerability science or how biophysical changes are experienced and responded to  Results show a "preference for the present" (p.817) - need to do more projections; more adaptation research; more focus on non-harvesting sectors as impacted by climate change. | Record resembles another paper by the same authors: same search; very similar framing; different types of results. In this case, it is focused on the state of knowledge on human dimensions rather than health impacts. Consider in conjunction with the very similar article noted, which also described impacts in detail.  Barriers noted to incorporating TK into resource management; and tension between "scientific understandings of 'adaptation planning' and Inuit understandings of 'planning' which may inhibit the effective collaboration of scientists with community members" (p.814) | Community-level strategies: Commonly identified determinants of adaptability/resilience include TK, social learning, flexible institutions, strong community social networks, acceptance and experience with uncertainty, and government support.  Future research: (i) Geographic disparities indicate that greater attention should be paid to regional centers and communities neglected by climate change research, along with a focus on vulnerable sub-groups; (ii) Research should consider the risks and opportunities posed by climate change to economic sectors and Inuit health, alongside the largely harvesting-focused studies conducted to-date; (iii) There is a need for regional analyses alongside local case studies; (iv) The risks posed by projected future climate change need to be examined and factored into decision making; (v) Scholarship would benefit from broader engagement of disciplines; (vi) There is a need for research to address how processes operating on multiple scales affect vulnerability and constrain or enable adaptation; and (vii) Greater focus is needed on identifying and characterizing adaptation interventions at multiple levels that are feasible, durable, equitable, and are sustainable in the long-term. |
| Gupta et al. (2022). Community-based responses for tackling environmental and socio-economic change and impacts in mountain social–ecological systems. Ambio, 51(5), 1123-1142. | Climate change is associated with increased frequency of extreme events; loss of snow cover; drying up of water resources  Climate change is "one of the most potent and visible drivers of environmental change in mountainous contexts" (p.1129)  Many mountain SES experience change through over exploitation, land use change, and land abandonment, and the vulnerability these forces creates for SES ecosystem degradation, biodiversity loss, and TLK are exacerbated by climate change | IPSI case studies reviewed established that community engagement is critical to the development and implementation of community-based interventions in mountain SES, arguing that local communities should be involved in conservation and sustainability planning to strengthen community engagement.  Introduction situated this work in helping to respond to/achieve SDG 15.4 on the "conservation of mountain ecosystems and their biodiversity"  Results on 'responses' to these changes in SESs included: mobilization of TLK, "biocultural databases", local TLK platforms, promotion of traditional land use practices, re-establishing keystone species, conservation-related projects; preservation/documentation of TLK & practices, knowledge-sharing; capacity building, awareness-building, participatory planning/co-management (p.1133)  Fig.7 (p.1135) a very interesting visual - interconnectedness of a variety of concepts | Research or policy: need to consider "both social and ecological dynamics" when determining responses to changes in mountain SES; one-size fits all approach not appropriate.  Policy: a combination of response options needed (rather than single responses), e.g., both local and national legislation to protect/conserve regions.  Community-level: local communities are key in terms of response; strengthen community engagement/empower communities, prioritize local-level decision-making; involve local communities in monitoring & evaluation of interventions  Need to develop “biodiversity and TLK registers”/documentation as means of improving community participation and restoring lost or dying TLK. |
| Markkula et al. (2019). A review of climate change impacts on the ecosystem services in the Saami Homeland in Finland. Science of the Total Environment, 692, 1070-1085. | Snowbeds, snow patches and mountain birch forests are particularly vulnerable to climate change impacts.  All the ecosystems treated in this article are also cultural landscapes, and changes in these ecosystems will alter peoples' sense of place and erode cultural meanings, stories, memories and traditional knowledge  attached to them.  Alterations in ecosystems affect the traditional Saami livelihoods, such as reindeer husbandry, salmon fishing, gathering, ptarmigan trapping and Saami handicraft, which consequently leads to a loss of terminology and practice-based traditional knowledge. Moreover, regulating and supporting services provided by the threatened ecosystems will be altered. These include climate regulation, nutrient cycling, soil formation and photosynthesis. |  | Both scientific knowledge and observations of local people about weather and nature conditions are valuable, since climate warming together with several other drivers has a direct impact on different ecosystem services, such as the traditional livelihoods. When local/traditional knowledge and scientific knowledge are combined, it is possible to arrive at both holistic and in-depth understandings of the interaction between nature and humans. |
| Borish et al. (2022). Relationships between Rangifer and Indigenous Well-being in the North American Arctic and Subarctic: A Review Based on the Academic Published Literature. Arctic, 75(1), 86-104. | Need to know more about the emotional connections that arise from Indigenous-Rangifer linkages, the effectiveness of adaptive strategies, and the intergenerational implications of Rangifer-related change.  Enhanced inclusion of Indigenous Peoples in the production of knowledge on this topic is fundamental to the future of understanding Indigenous-Rangifer relationships. | Focus is not squarely on the impacts of biodiversity loss (specifically Rangifer), though these are mentioned and implied. Rather, the focus is on characterizing the connections between Rangifer and Indigenous Peoples' wellbeing more broadly. | Future research: need to know more about psychological/emotional wellbeing related to Rangifer (so mental health planning can be supported); influence of change in species on Indigenous wellbeing; how communities are adapting; how knowledge transfer to younger generations is happening (what are short/long-term implications if youth cannot access/learn about Rangifer). Also need to understand dietary substitutions for Rangifer and influence on well-being; clearer reporting/publication standards for explicitly discussing Indigenous Peoples' engagement in wildlife-related research. Finally, need to Increase partnerships/collaboration between researchers and Indigenous Peoples, organizations, governments; increased need for co-production of knowledge on Rangifer-related change, embedded in partnership.  Community-level: "Strategies to support Rangifer-specific intergenerational knowledge transmission within communities" (p.98) |
| Charnley et al. (2022). Drought-related cholera outbreaks in Africa and the implications for climate change: a narrative review. Pathogens and global health, 116(1), 3-12. | Drought frequency/patterns may alter with climate change, spatially heterogenous changes in drought (e.g., El Nino more frequent, abnormally hot/dry, impacts droughts)  Unpredictable rainfall means water availability will be increasingly complex, potentially leading to increased risk of cholera  Inequitable impacts of drought due to climate change (poorer, rural communities at heightened risk of cholera, "due to reliance on agriculture and inability to afford alternative safe water sources") | Only based on 6 articles; most of the information above is drawn from the discussion, not the results of the synthesis itself.  Drought is framed as a health outcome but was not defined on how it is quantified (start and end) | Policy/public health level: important to reduce other vulnerabilities (socio-economic, etc.) in order to prevent outbreaks, minimize impact of drought. Could include enhancing coverage of WASH; alleviating poverty and reducing marginalization more generally. Additionally, more self-sufficiency of countries in disaster prevention; improved multi-national agreements for sharing/managing natural water sources and drought response  Future research: tools for enhanced surveillance related to drought/ability to prioritize areas for cholera prevention |
| Pearce et al. (2011). Advancing adaptation planning for climate change in the Inuvialuit Settlement Region (ISR): a review and critique. Regional Environmental Change, 11, 1-17. | The literature identifies a number of barriers and limits to adaptation, which are undermining adaptive capacity, and provides insights for future vulnerability.  Damage to municipal infrastructure also caused by climatic conditions, such as built infrastructure being sensitive to permafrost thaw as well as transportation infrastructure being vulnerable to coastal erosion, floods, and warming in shoulder seasons and threatening the integrity of buildings and services. | Socioeconomic drivers to adaptability or vulnerability also discussed (e.g., capital resources) | Community/population level: adaptation strategies include switching species of wildlife harvested, development of youth-Elder mentoring programs to facilitate traditional knowledge/skills transmission (often autonomous to individual/household level and reactive in nature). Diversity of practices to cope also include livelihood and economic diversity to spread risks, traditional knowledge allowing exploitation of risky environments and management of dangers, social networks through which risk is shared  Policy level: The above factors are policy entry points for adaptation planning (e.g., supporting land skills and environmental knowledge transmission among younger generation would enhance adaptive capacity to current/future climate change). Adaptation planning should be focused on community level and have engaged community stakeholders  Future research: It’s important that research also examines how projected changes might interact with biological and human systems to affect exposure-sensitivity and how socio-economic-demographic trends will affect how communities experience a changing climate. Location-specific assessments of climate effects would improve understanding of future vulnerability. Research has tended to focus on physical impacts of climate, and needs to expand on interactive effects with other social, cultural, and economic processes which influence how communities experience climate change. |
| Davis et al. (2022). Shifting safeties and mobilities on the land in Arctic North America: a systematic approach to identifying the root causes of disaster. *Sustainability*, *14*(12), 7061. | High cost of living in the Arctic forces costs to be high (impacted by historical/contemporary environmental policy)  "Rather than climate hazards being primary drivers of risk in Arctic North America, root causes embedded in colonialism, in the context of changing weather and climatic conditions, are responsible for creating barriers to safe travel on the land" AND "the unpredictability of weather conditions becomes a greater problem in the context of these root causes and drivers of risk, and these risk drivers act as a barrier to the exercising of the flexibility and life-long learning that Inuit are able to employ" (p.13) | FYI-The focus of this paper is not climate/environmental change as the driver of mobility constraints (just that these constrains occur in the context of a changing climate).  "Role of climate change was discussed in some studies within this review, though often in a non-direct way, or as one of a number of factors" (pg.10)  pg.2- "[for Inuit in Arctic North America] the land is the heart of cultural and community life"  pg.2 - "Inuit relationships to land require flexibility in the face of constant change and dynamic uncertainty in the Arctic, and Inuit Knowledge provides this"  pg.2 - many more important concerns to Inuit than climate change (also echoed in another article we reviewed) | Policy-level: Increased social protection, and movements toward greater Indigenous autonomy over mobility, time, education and land use are ways to tackle the root causes of risk.  Future research: more detailed analysis of the specific institutional and governance processes which are presently replicating and recreating structural violence and processes of risk. "As Hall and Sanders [69] suggest, research that centres on climate change as a threat to Arctic Indigenous Peoples fails to reconsider the political and legal institutions and structures that constrain Arctic resident’s ability to adapt to climate change. . .to see resource extraction and shipping as human dimensions of climate change. . .and to recognize that hunting policies set in Washington, London, and Brussels may pose a greater threat to Arctic citizens than the changing biophysical climate”(p. 443)  Broadly: to recognize the broader changes to socio-political systems required to adapt to climate change, and which create the conditions of risk and constraints to adaptation for communities (see bottom of pg.14 for very important/relevant take-aways! e.g., "There is…value and opportunity in using a climate change lens in a nuanced way to reveal the socio-political structures and inequalities that underly climate change experience [222,223] and to leverage action and seize a window of opportunity, providing it does not distract from the underlying, socially located root causes of risk [97]." (p.14); "there needs to be a shift from a focus on “vulnerable peoples” to the underlying processes and institutions that put people at risk" (p.15) |
| Ford et al. (2012). Research on the human dimensions of climate change in Nunavut, Nunavik, and Nunatsiavut: a literature review and gap analysis. Arctic, 289-304. | As shown in this study and elsewhere, HDCC research is a rapidly expanding field. The growth in regional interest in the Arctic is an important development and holds potential to inform communities and policy makers about the risks posed by climate change and the prospects for intervention to reduce its negative effects and take advantage of new opportunities  "The Arctic, as a "miner's canary" of climate change, is also an early warning opportunity to increase our understanding of how climate change will play out in other regions" (p.298) | Vulnerabilities described, and then associated adaptations for infrastructure challenges, including changing timing/location of harvesting activities; switching species; new travel routes; avoiding travel at certain times/locations (knowledge of hunters critical; knowing precursors to hazardous conditions; how to prepare & survive); technology (e.g., geo-coded Inuit place names via GPS; satellite phones; VHF radios; distress beacons)  Also mentioned community-based observation/monitoring networks | Policy level: strengthening food systems by investing in/enhancing harvester support programs; community freezers/food banks; youth hunting programs; meat sharing initiatives  Future research: need for more "action-oriented HDCC projects [which promote]...community-based participatory research approaches" (p.299). Also need "to develop a broader and more diverse geographic and sectoral knowledge base".  Research & policy: many questions noted (p.299) around how future social/economic/political changes will affect Northerners; how vulnerability and adaptation will look in future; roles of government in supporting adaptation; how adaptations should be assessed and by what criteria, etc.? |
| Lam et al. (2019). Community-based monitoring of Indigenous food security in a changing climate: global trends and future directions. *Environmental Research Letters*, *14*(7), 073002. | The increasing interest in CBM is part of a broader trend around need to address the disproportionate climate change impacts experienced by Indigenous communities globally. | Climate change indicators were rarely assessed/including in community-based monitoring activities reported in reviewed articles, which could be explained by lack of consensus on how it should be assessed related to food systems resilience.  Discussions not explicit to indigenous health | Future research: incorporating Indigenous knowledge in design of climate change metrics to help improve understanding of how climate change impacts on food systems can be assessed over time. Also need for development and inclusion of climate change metrics in community-based monitoring are recommended, and greater attention to autonomous monitoring and adaptation could help inform more effective responses to climate change.  Policy-level: importance of considering indicators of wildlife and environmental change in food security monitoring systems, considerations that are typically excluded in conventional food security monitoring efforts. |
| Ford et al. (2010). Vulnerability of Aboriginal health systems in Canada to climate change. Global Environmental Change, 20(4), 668-680. | Relationships studied are framed around SDOH (economic poverty, technological capacity, socio-political values/inequality, constrained institutional capacity, information deficit) that increase the susceptibility of IPs to climate-related poor health outcomes, as well as pose as major constraints to adaptive capacity.  Mitigation is essential, but health systems are going to need to adapt to manage climate-sensitive health outcomes  Overall uncertainty as to climate change impacts on Indigenous Peoples' health; "constrains effective risk assessment and planning" (p.677) | The results of this paper are based on sensitivities/broader determinants of climate vulnerability for Indigenous Peoples, not impacts, such as context for increased sensitivity of Indigenous Peoples to climate change/health effects: higher existing burden of ill-health; higher dependence of livelihoods on environment; spiritual/cultural ties to land  Other broad determinants of vulnerability, which increase Indigenous Peoples' sensitivity to climate-health risks (pgs.673-677) including: (1) poverty (overcrowding; inadequate housing; food/water insecurity; broader psychosocial conditions of poverty limit adaptive capacity); institutional constraints in human & financial resources, constrained capacity for health systems to adapt/assess/plan for climate change impacts (2) accessibility of health services (small, remote communities) & availability of technology for health systems; insufficient surveillance systems to detect occurrence/spread of climate-health risks; high staff turnover (3) political inequality linked to range of negative social/economic/health outcomes;  (4) unclear jurisdictional responsibility with respect to health service delivery; limited accountability/conflict/complex bureaucracy that constrains ability to adapt/plan/prepare to climate-health risks. | Policy level: need to improve access/reduce inequalities to health services - important to climate change vulnerability (includes collaboration between Indigenous Peoples; policy makers; frontline health workers RE: cultural sensitivity; training of practitioners in traditional & Western health systems; improved technology to communicate health information) -need to build more effective public health surveillance systems with "culturally specific and locally relevant health indicators" (p.675) -increased participation of Indigenous Peoples in decision-making/accountability; financial commitments relevant to climate change adaptation/mitigation; open dialogue to protect, respect, fulfill rights -"A new ethos of coordination and cooperation among government levels" (p.677) in order to address institutional determinants of poor health and build health systems that are self-reliant, effective, and adaptable. -need for ongoing, collaborative vulnerability assessment at local-regional scales to characterize sensitivities and evaluate health planning.  Future Research: interdisciplinary research that "characterize[s] climate vulnerabilities specifically at local and regional levels and identif[ies] and prioritize[s] opportunities for adaptation" (p.677). |
| Loring & Gerlach (2015). Searching for progress on food security in the North American North: a research synthesis and meta-analysis of the peer-reviewed literature. *Arctic*, 380-392. | Authors argue that food security (in 2015) was more a political and social problem rather than bio geographic or ecological - since access to food is the keystone identified in their analysis. "While there is no doubt that climate change and the unique circumstances of living in the North play a role, they only serve to exacerbate more fundamental drivers: top-down governance structures, inflexible policies regarding land use and resource management to environmental change, and economic development agendas that marginalize the rights and needs of Indigenous peoples"  Loring and Gerlach (2009), for example, argue for an integrated, social-epidemiological approach to food security that links biophysical, psycho- logical, psychosocial, and sociocultural dimensions of food systems. Similarly, Gadamus (2013) describes direct and indirect reciprocal relationships among ecosystem health, food security, and human health in the North  Indigenous people in the North are largely locked in "Manufactured insecurity" | Authors draw on need to understand context of socio-economic transformations affecting Inuit society and affecting food (in)security experiences | Policy responses: ideas for strengthening self-governance in the North, therefore, need to see beyond concepts such as adaptation and resilience and look to rights-based reform (such as food sovereignty).   Future research: there is a gap of community-based participatory research and other participatory/Indigenous approaches, which could align with rights-based approaches deserving of increased attention in academia and policymaking. "Even the ability to define the problem on one's own terms represents in many places a move away from the status quo" |
| Ford (2012). Indigenous health and climate change. American journal of public health, 102(7), 1260-1266. | Climate change impacts occurring faster and of greater magnitude for many Indigenous populations.  Significant focus in the article on adaptive capacity of Indigenous populations: community-based education/awareness initiatives; research that empowers communities; etc.  "Impacts, adaptation, and vulnerability are highly place- and culture-specific, and mediated by worldviews that place significant value on interpersonal and environmental relationships, stewardship, life experience and balance, spiritual considerations, family, tradition, and oral history." (p.1263) | Broader context for increased sensitivity/exposure to climate risks connected to social determinants of health for Indigenous Peoples (poverty; burden of ill health; overcrowding; access to education and employment). Also linked to hydrocarbon-related resource development; deforestation and challenging access to/ownership of traditional lands; already high burdens of disease; discrimination when accessing formal health services, etc.  Broader determinants of socioeconomic and health inequality that lie at the heart of indigenous vulnerability to climate change: Colonialism; colonial history, assimilationist policies, land claim disputes, limited autonomy, social/political violence | Community-level: adaptation strategies "underpinned by traditional knowledge of lands and resources, cultural identity, and strong social and kinship networks, within health systems that combine allopathic and traditional approaches."  Research & policy: "how global processes interact with local conditions to create vulnerable (or adaptable) populations in light of a rapidly changing climate" (p.1263). "Indigenous conceptualizations on and approaches to health need to be articulated and central to research" (p.1264). Further, more baseline data on socioeconomic and biomedical determinants of vulnerability, the sensitivity of health outcomes to climate and SDOH; research that involves "an evaluation of opportunities for policy intervention"; interdisciplinarity. Need to develop and improve surveillance and environmental monitoring systems", particularly those that are grounded in local capacity/activities (p.1264). Finally, develop and evaluate adaptation interventions, e.g., "efforts to preserve, document, and promote and teach traditional knowledge among younger generations and strengthen links to traditional activities" (p.1264) |
| King & Furgal (2014). Is hunting still healthy? Understanding the interrelationships between indigenous participation in land-based practices and human-environmental health. International Journal of Environmental Research and Public Health, 11(6), 5751-5782. | Health of people and place demand an integrated engagement to embrace a more holistic view through development and use of new, transdisciplinary methods has considerable merit. The end goal for Indigenous people of being able to realise any benefits of ongoing land-based practices can only be achieved if the health of the environment is seen as integral to, not separate from, that of the people in it. |  | Research & Policy: How “health” is understood requires a considerably broader lens than that focused predominantly on individual human biology. To do this requires changing perspectives in the way research and policy makers approach these complex issues. Through transdisciplinary approaches the current gaps and assumptions about the complex interrelationships between land and human health in Indigenous contexts become understood and sustainably addressed by moving beyond disciplinary and culturally bounded spaces. |
| Dannenberg et al. (2019). Managed retreat as a strategy for climate change adaptation in small communities: public health implications. Climatic change, 153, 1-14. | Sea level rise associated with climate change, creating a pathway to "acute and gradual reductions in habitability" (p.7); Overlaps with erosion, storm surge, tsunami risk, saltwater intrusion, ecosystem damage and prompts managed retreat, which has associated health impacts.  Health impacts of relocation framed as "indirect", related to "physical and social determinants of health" (e.g., housing, source of livelihood, social capital, cultural identity and traditional knowledge, land security, access to energy, social support services, education, coastal access, familiarity with a new location, room for population growth, conflict with other populations). | None of the included case studies have explicit measurements of health impacts of managed retreat ("qualitative suggestions of health impacts", p.8)  Reference at end of p.8 to Indigenous Peoples who may be particularly vulnerable because they already have poorer health/fewer resources, but also have a “strong tradition of intergenerational stewardship of and responsibility for the land” (McMichael et al. 2010) as a source of strength. | Policy level: "structured frameworks and tools may facilitate relocation processes"; need to prioritize the "rights of communities to adequate health and other services during and after the relocation process"; precautionary relocation efforts are important (most successful when community voluntarily initiates it); bring a 'cost-benefit analysis' approach to management discussions and resettlement dialogue.  Health sector: grief counselling available to people  Future research: the sociocultural and health implications of managed retreat deserve substantial attention" (p.12). How to facilitate "population resilience before, during, and after relocation" (conduct surveillance of health indicators before, then after relocation to assess impacts); comparison of health impacts of relocation from sea level rise as compared to other reasons for migration. |
| Kipp et al. (2019). The need for community-led, integrated and innovative monitoring programmes when responding to the health impacts of climate change. International journal of circumpolar health, 78(2), 1517581. | Review found several important opportunities that exist with regard to Indigenous communities monitoring the health effects of climate change, where community programs need to: 1) be community-led and -based; 2) holistically incorporate health and environment; and 3) make use of innovative tech that are locally appropriate and usable -- which may explain why environmental indicators were more frequently monitored than health indicators.  Few community-led programs were identified, despite all programs reporting some level of community involvement. | Challenges associated with collecting health indicators included 1) identifying relevant indicators dependent on populations vulnerability and adaptive capacity (and other determinants), 2) accessing/analyzing data from multiple sources is difficult, 3) determining relationships between complex and distal climate risk factors and health consequences is methodologically challenges, and 4) gathering/analyzing data requires resources/capacity. | Future research: community-based monitoring programs should aim to be led by community members, fill gaps in monitoring health implications of climate change, and use innovative solutions/upscaled tech to gather data and monitor indicators. |
| Jaakkola et al. (2018). The holistic effects of climate change on the culture, well-being, and health of the Saami, the only indigenous people in the European Union. Current environmental health reports, 5, 401-417. | Lifestyle, diet and morbidity of Saami is changing posing threats to physical and mental health. Climate change brings environmental, economic, cultural and social effects on reindeer herding as both an economical livelihood and cultural way of life. Cultural wellbeing in the Saami context depends on social community and kinship structure, environmental relationship, and traditional livelihoods including language and other aspects of culture. | Melting of sea ice and opening of new resources for extraction increase risk of environmental accidents and increase land-use competition, limiting climate change adaptation for Saami to maintain their traditional livelihoods. Small population size, dispersed settlement and possibility for increased urbanization limiting cultural adaptation in the face of climate change.  Record reports that most studies characterize environment, culture, livelihoods and health without any consideration of role of climate change.  Focus of the record's identified impacts is also on reindeer and Saami people together.  Record also notes that socio-economic challenges not only threaten Saami culture and identity maintenance, but weaken possibilities of Saami to adapt to climate change.  Finally, author's Figure 5 and Article Tables (2&3) may report more findings in its summary relevant to our review than their Results section actually reports - particularly distal impacts. | Community/population-level: Adaptation in reindeer herding to adverse climate effects, such as flexible use of pasture land, use of environmental knowledge, provide supplementary feeding. Reindeer can also be seen as tool for climate adaptation, such as grazing to protect tundra biome from shrubification and tree line encroachment. Incentive grazing can also increase surface albedo in summer, delay snowmelt, and decrease the ground from heating in snowmelt season.  Future research: A new assessment of the effects of climate change to Sapmi and Saami culture and a projected outlook for the future are needed. Also, more research and development of a monitoring system necessary to understand the health and cultural wellbeing of Saami in a changing climate taking into consideration Saami values and traditions and major drivers for change. |
| Jasmine et al. (2016). Traditional knowledge systems in India for biodiversity conservation. | India is making a number of strides in terms of policies, programs, plans, and other initiatives, to document and apply TK across the country. They feature integration at the national and regional level for strengthening countrywide collaboration, mediating interventions, and interpreting policies recognizing importance of TK in conservation of biodiversity and human well-being.  The sustainability of traditional ecological knowledge to the environment (including socio-ecological systems) are needed. | The article only touches on environmental impacts on biodiversity, specific to changes in socio-ecological and cultural systems of knowledge transmission.  Note: no health or wellbeing impacts or how these strides are affecting biodiversity or Indigenous/local peoples are mentioned in the record. | Policy-level: Management of India's TK database to further examine changes in ecosystems and the resources and services they generate, along with highlighting the role of local or Indigenous communities in using TK. Local communities could therefore gather further knowledge and network with other regional communities. Reviewing the nature of the Aichi Biodiversity Target 18 and Indian's National Biodiversity Target 11 to address issues of importance of TK for communities to protect and develop equitable distribution of economic benefits.  Future research: link the sustainability of TK to the environment, amalgamation of knowledge and findings to socio-ecological systems needed through sourced information from a wider range of stakeholders. Evaluating the Indian system, and understanding TK scope in agriculture and medicine, for qualitative and quantitative factors also needed to be able to integrate management of resources and ecosystems should be considered to enable local knowledge use and livelihoods. |
| Hillier et al. (2021). Examining the concept of One Health for indigenous communities: A systematic review. One Health, 12, 100248. | One Health framework was reported as a tool to study the health effects of climate change because it centres community consultation to identify resident priorities & solutions, as well as prizes traditional knowledges through this consultation.  "One Health was described as a potentially useful framework for interventions addressing climate change-induced health problems" (p.3) and a "tool to study the health effects of climate change" (p.3)  One Health approach "prioritizes traditional knowledges through community consultation" (p.3) and attempts to address the causal role of climate change in relation to 'downstream determinants'. Also has "strong correlation with established Indigenous knowledges and practices" so should be applied when working in collaboration with Indigenous Peoples (p.4). | Article is arguably more focused on research approaches/methodologies (more conceptual). | Community consultation/international collaboration important when it comes to One Health interventions (and this type of multi-stakeholder engagement is considered a tenet of the One Health approach itself)  Future research: One Health approach could be used for improved disease surveillance; a "holistic and interdisciplinary approach to address the resulting health concerns for Indigenous Peoples, biota, and the environment" (p.4). There’s a need to more foundationally integrate Indigenous knowledges/worldviews into One Health; not consider it an 'add-on' to the framework. |
| Eerkes-Medrano & Huntington. (2021). Untold Stories: Indigenous Knowledge Beyond the Changing Arctic Cryosphere. Frontiers in Climate, 3, 675805. | Climate change is not the whole story as human responses have the great scope to determine the ultimate outcomes of these "untold stories" of a changing cryosphere. Government policies have more impacts sometimes than climate change. The “changing Arctic cryosphere has not been fully explored" (p.2) - from perspectives of Arctic Indigenous Peoples or the implications of the changes for them.  Part of the story of the Artic Change is being told, but part remains obscured or are missing. “Perspectives of Arctic Indigenous Peoples as reflected in these publications often suggest a degree of hope that is typically missing in visitor’s assessments of the prospects for Arctic communities in a changing climate" (p.2) Also that the negative impacts/challenge of change is not the whole story, but that the included articles "suggest a different narrative, one emphasizing connection and response, rather than inevitability and loss." (p.13)  "Our review has shown that climate change is not always the primary threat to Indigenous well-being in the Arctic, one spelling doom for Arctic Indigenous peoples." (pg 14) | More about adaptation than specific health/wellbeing impacts, largely.  Arctic cryosphere "regarded as one of the most visible signs of global warming, as sea ice retreats, snow decreases, glaciers and ice caps melt, and permafrost thaws...far-reaching implications for biology and society in the Arctic and beyond" (p.1-2). While changes are serious and pose a major threat, necessitating adaptation, Arctic communities “have long cultivated the flexibility to deal with that variability" (p.6)   Discussion that "recent environmental change is seen by many Indigenous persons as simply one of many forms of change they are experiencing" (p.12), among broader changes to commercialism/economic developments, social and political changes (land claims/sovereignty; creation of local governments); technologies/communications in the Arctic.  Ability to move freely has been key to adapting to changing conditions for many Arctic Indigenous Peoples (can be limited by government regulations and infrastructure) (p.13) | Policy-level: a call from included papers for two-way "partnerships in solving problems [e.g, with government agencies/scientists] rather than a request to have problems solved for Arctic communities" (p.6), same with bilateral sharing of information between scientists and communities rather than "providing unilateral advice" (p.6), Also, or IPCC to continue to partner with Indigenous Peoples to identify thematic gaps and regional gaps in coverage; allocate necessary time/funding to these areas.  Community-level: "continued learning from one another as individuals and communities in the Arctic to develop new ways of doing things." (p.6)  Future research: mental health impacts of a changing cryosphere; assessing state of knowledge on cryosphere-related impacts in other Arctic Indigenous communities, beyond Inuit (p.6); specific changes to permafrost (p.11); more research generally in "how a changing cryosphere affects community and individual well-being" (p.11) and research that "make[s] an explicit connection between Indigenous knowledge, a changing cryosphere, and the full range of today's activities and concerns in the Arctic" (p.12). Also, more research on personal stories of the changes people are experiencing and what those changes mean for the individual and their community (some specific stories on pgs.12-13)  Broadly: "It is particularly important that Indigenous voices are heard and that Indigenous ideas are part of the discussion about Arctic change and its meaning" (p.13) |
| Little et al. (2021). Drivers and health implications of the dietary transition among Inuit in the Canadian Arctic: a scoping review. Public health nutrition, 24(9), 2650-2668. | Climate change is a key driver in dietary changes among Inuit because of its impositions on land access and the safety/security of country foods. |  | Future research: need to prioritise research on impacts of climate change on accessibility, availability and affordability of CF and MF and potential adaptation strategies to mitigate the risks of climate-mediated diet-related risks to Inuit health and well-being and integrate these issues in the risk-benefit analyses. |
| McNamara et al. (2021). Exploring climate-driven non-economic loss and damage in the Pacific Islands. Current Opinion in Environmental Sustainability, 50, 1-11. | 3 main health trends: 1) health impacts are experienced individually but they impact Pacific Society broadly; 2) rising climate-induced mental health impacts; and 3) the disproportionate health impacts on women, children, and elderly  Human mobility was the most prominent theme discussed, along with culture and cultural heritage loss. Health impacts are experienced individually but they impact Pacific society broadly. Rising climate induced mental health impacts, losses to biodiversity and sense of place. | Interesting comment about evidence of varied and tighter types of social connections and more diversified networks being reinforced during times of stress as people support each other. | Future research: Lack of studies that look at losses of Indigenous knowledge; few studies that address biodiversity and ecosystem services loss along with explorations of grief and mourning. Sense of place as another area of exploration and its overlap with health issues, particularly in relation to 'solastalgia' - distress produced by environmental change impacting people while the are directly connected to their home environment. Important to document losses as well as strengths to identify ways of minimizing harm. |
| Jones et al. (2020). Climate change mitigation policies and co-impacts on indigenous health: A scoping review. International Journal of Environmental Research and Public Health, 17(23), 9063. | Sharing of power, both in the research process and in the conception, design and implementation of climate policy and interventions, will be essential for Indigenous rights and health equity  Most climate policy studies did not consider Indigenous health equity, nor use Indigenous or decolonial research methodologies to shape the questions/outcomes/interpretations/actions.   Climate change mitigation policies can produce both co-benefits and co-harms, and adverse equity impacts of climate policies can be mitigated only deliberately and with community engagement. | Authors note that the impacts of interventions reviewed on Indigenous health had varying mechanisms, so no discussion or clarity on what/how/why these interventions produce the discovered co-benefits or co-harms. | Future research: partnership with Indigenous communities and privileging IK should be used to improve the quality of evidence about co-impacts of climate policy on Indigenous health to inform pro-equity climate mitigation. It’s also possible to improve the quality of evidence about co-impacts on Indigenous health in order to inform pro-equity climate mitigation by encouraging partnership with Indigenous communities, recognition and privileging of Indigenous knowledges and study design that fully embeds Indigenous values, realities and priorities. |
| Bryson et al. (2020). Neglected tropical diseases in the context of climate change in East Africa: a systematic scoping review. The American journal of tropical medicine and hygiene, 102(6), 1443. | A changing climate impacts geographical range and incidence of NTDs; parasitic habitats/life cycles regulated by environmental conditions, as are vector lifecycle and transmission of pathogens  "Temperature impacts human behaviour involved in disease transmission - can modify exposure to some NTDs" (p.1449)  A country's financial position affects its ability to mitigate disease outbreaks and adapt to climate changes. | Also found that the countries with highest publication counts in region (Kenya, Tanzania, and Ethiopia) vs lowest publication counts (Comoros, Madagascar, Malawi, Mauritius, Mozambique, Seychelles) shows disparities in financial incomes and international economic assistance which benefit from research to understand status of NTDs. Lower income countries also have higher risk of negative consequences from disease and climate change because of limitations on financial resources and adaptive capacity.  Indigenous Peoples identified in few publications. | Future research: Further research needed to "understand how NTDs are affected by climate and extreme weather in East Africa" (and changing climate, as implied). Also needed on vector-borne diseases with a high burden of disease in East African countries and which are climate sensitive (projected to increase with climate change across African continent): schistosomiasis, lymphatic filariasis, visceral leishmaniasis. Further research also needed on unique challenges of Indigenous Peoples in relation to climate change and impacts of NTDs; as well as examining social/cultural contexts that influence NTDs alongside more equitable regional representation in research that examines climatic factors and NTDs. Finally, need for research to equip public health institutions with respect to disease management. |
| Vogliano et al. (2021). Progress towards SDG 2: Zero hunger in melanesia–A state of data scoping review. Global Food Security, 29, 100519. | Findings suggest that traditional agrobiodiversity and food system knowledge are being lost to urbanization, lifestyle changes, imported foods, and deforestation.  Indigenous Melanesian food systems is declining, related to crop diseases and pests, climate change, deforestation, and industrially produced monocultured crops such as palm oil – all of which are disrupting traditional agri-food production and associated knowledge. |  | Future research: the conservation of bees and associated ecosystem services are critically important yet understudied component of sustainable food systems and require additional research. Further, participatory knowledge production should be used to build trust with Indigenous Peoples and address barriers such as cultural differences, inappropriate governance structures, and inadequate technical and political support.   Community & Policy levels: Recommendations include education around seed saving and establishing community seed banks and plant collections. Equipped with such knowledge, farmers in this biodiversity hotspot may maintain their traditional plant heritage, diversify their diets, and improve their livelihoods. |
| Ingemann et al (2020). Patient experience studies in the circumpolar region: a scoping review. BMJ open, 10(10), e042973. | Contextualizing climate change as determinant of patient experiences would necessitate broader definition of a "health system", shifting a framework to a values-based conception grounded in circumpolar context and Indigenous knowledge. | Climate change discussion is very minimal in this article - only 3 articles (of 96) discussed it, all from Canada, and discussed alongside importance of Indigenous values. Climate change also only came up as a factor in qualitative and mixed-methods studies. | Future research: qualitative methods used to develop and contextualize a circumpolar patient experience framework, which would reflect what is important to patients and guide future studies in the area of patient experience in this region. Future studies should also focus on how health systems can become more responsive to the needs of circumpolar populations.  Policy level: redefining "health systems" to integrate Indigenous ideologies, and climate change, as determinants of patient experiences. |
| Middleton et al. (2020). Indigenous mental health in a changing climate: a systematic scoping review of the global literature. Environmental Research Letters, 15(5), 053001. | Climate change is driving widespread mental and emotional impacts. | Mental health-climate experiences by age, such as youth and Elders or older community members (latter identified as vulnerable for mental health impacts). Youth expressed concern for Elders and seniors, too. | Future research: application of diverse methodological approaches and increased engagement with Indigenous communities will be necessary to document intangible climate-driven impacts, determine the potential limits of adaptation, and propose desirable alternative pathways |
| van Bavel et al. (2020). Contributions of scale: what we stand to gain from Indigenous and local inclusion in climate and health monitoring and surveillance systems. Environmental Research Letters, 15(8), 083008. | From the review, synthesis, and confidence assessment of integrated climate-health monitoring and surveillance literature, we found that the inclusion of diverse knowledge systems contributes to these systems through the collection of more representative data; the reduction of selection and source biases; the processing and interpretation of more comprehensive datasets; as well as immediate decision making and prioritization of key issues. | This is a unique review article that not only provides a summary of review findings, but provides a synthesis in evidence on the inclusion and contributions of diverse knowledge systems to integrated climate-health monitoring and surveillance systems. | The inclusion of diverse knowledge systems contributes to integrated climate-health MSS through the definition of meaningful problems; the collection of more responsive data; the reduction of scale dependent biases; the development of multi-scale policy; long-term future planning; as well as creating effective knowledge-information-action pathways. Lastly, the inclusion of diverse knowledge systems contributes to integrated climate-health MSS through the divergence and discordance of methodologies and evidence. |
| Akearok et al. (2019). Identifying and achieving consensus on health-related indicators of climate change in Nunavut. Arctic, 72(3), 289-299. | "Environmental changes can directly or indirectly impact human health by affecting the stability and predictability of the natural built environments on which humans rely for positive health outcomes"  Pre-existing "population vulnerability" modifies/focuses these impacts differently (e.g., pre-existing chronic disease in Arctic; poverty status) | Clarifying that this article discussed impacts, but the focus was on developing/prioritizing INDICATORS for monitoring/evaluation specific to the Canadian Arctic. | Future research: need mitigation, adaptation, and policy indicators to measure the public health efforts involved in response to climate change, and research evaluating the usefulness in practice of indicators identified. It’s important to explore barriers that might exist to why a given community wouldn't have climate change initiatives in place. Research on community-led approaches/interventions also needed.  Policy: it is crucial to continue to involve local communities in monitoring/adaptation. Also need to continue to have cross-sector/multidisciplinary/societal collaboration to mitigate health effects of climate change |
| Sahu et al. (2022). Measuring Impact of Climate Change on Indigenous Health in the Background of Multiple Disadvantages: A Scoping Review for Equitable Public Health Policy Formulation. Journal of Prevention, 1-36. | Globally, many IP face higher exposure to climate change risks due to close relationships/dependence on natural resources and being remotely located.  They are suffering from a “triple burden of disease” due to the combined effect of chronic diseases, infectious diseases and climate change impacts where climate change impacts further exacerbate existing health inequalities. (Kumar et al., 2020). | Most articles focused on physical health effects, 5 on mental and pscho-social health aspects, one on health care access, and one on health stewardship.  Record speaks to socioeconomic (poverty, poor living conditions) and systemic (racism, poor health service access, living on marginal land) disadvantages experienced by IP which exacerbate vulnerabilities to climate change impacts.  Authors also identified local climate adaptation methods were reported in about half of records reviewed, which included impact measurement, health system assessment, and resilient community action from capacity building and resource management. | Policy level: climate mitigation policy must account for cultural and contextual factors of Indigenous communities in order to generate impacts, requiring a deliberate and planned approach to community engagement. Further, policies must be tailored to cater to specific health needs at a local scale using local data and expertise. Finally, climate solutions must be grounded in different ways of knowing, doing, being that reflect Indigenous values  Future research: improve the quality of evidence about co-impacts on Indigenous health to inform pro-equity climate mitigation, which requires partnership with Indigenous communities, recognition of Indigenous knowledge, and study design accounting for Indigenous values, realities, and priorities |
| Kiddle et al. (2021). An Oceania urban design agenda linking ecosystem services, nature-based solutions, traditional ecological knowledge and wellbeing. Sustainability, 13(22), 12660. | The critical need to better understand, and account for complex socio-ecological relationships when developing adaptation policies and urban development plans. Incorporating elements of traditional ecological knowledge and customary practices into nature-based solutions are likely to offer more acceptable, place-specific and long-term solutions in the Oceania context.  Two of many key concepts which have driven or influenced many Aoteaora matauranga Maori oriented projects include whakapapa (human genealogy lineages that trace back to landscape features including mountains and rivers and areas of ocean, and the origins of the beginning) and kaitiakitanga (an obligation through kinship to actively protect the mauri (essential life force) of living systems and waters).These considerations suggest to further investigation of the value that cultural knowledge, specifically matauranga, can add to NbS urban climate adaptation for enhanced human wellbeing in Oceania. Such investigation potentially offers benefits to the collective identity and health of the whole region. | Solastalgia may be particularly useful in describing the nexus between climate change, ecosystems and mental health and wellbeing for Pacific peoples. | Future research: Significant gaps and lack of data on how climate change affects mental health in the region, particularly as understood through worldview lenses of the people of Oceania. Also little investigation into how TEK is being defined and incorporated into EbA and other NbS or ecosystem-based initiatives. Significant potential for researchers and TEK holders to co-produce knowledge (and action) that is well placed to assist in climate change adaptation efforts that are more effective because they incorporate multiple knowledge systems, are more participatory and are culturally appropriate and affirming.   Policy level: Progressing nature-based forms/practices of urban design is important as part of wider efforts to adapt to climate change and protect and promote human wellbeing and the ecologies of the region. |
| Reis et al. (2022). Specific Environmental Health Concerns and Medical Challenges in Arctic and Sub-Arctic Regions. Health, (3), 22. | The climate knows no national borders  The Arctic and sub-Arctic populations are victims of the Anthropocene Epoch of human activity and the extension of a Westernized lifestyle that endangers local resources and indigenous cultures | Many of the health impacts described within this review relate to the population of this region more generally than specific to Indigenous Peoples. | Policy level: Increased cooperation among states is mandatory, as climate changes faster in the Arctic than elsewhere.   Further research: Bring better research and information to allow remediation and re-silience |
| Cottrell (2022). Avoiding a new era in biopiracy: Including indigenous and local knowledge in nature-based solutions to climate change. Environmental Science & Policy, 135, 162-168. | Authors of papers on NbS often are not mentioning/discussing concerns about "validation, integration, and protection of ILK" (p.166)  Most frequent discussion around decision-making with ILPCs, co-production of knowledge; and acceptance (recognizing the value/contribution of other knowledge systems to the issue).  Not much monitoring/evaluation of NbS projects; no discussion of sharing protocols (e.g., info-sharing between participants & researchers?); few examples of ILK protection (e.g., fair compensation for use of ILK in NbS projects)  "Potential for misappropriation and abuse of ILK could discourage participation by IPLCs" in NbS projects (p.166)  Legacy of "biopiracy" from scientific community, appropriating ILK  “Each of the 45 articles assessed in this study occurred in Indigenous and local communities and only around one-third of the articles mentioned IPLC participation or the inclusion of ILK" (p.167) | Different focus/framing of article - not speaking directly to impacts, rather the ways in which Indigenous Knowledge is positioned in relation to these broader movements in the scholarship (NbS) and global policy space.  Review also found examples where IP ecosystem management knowledge and strategies were unseated by outside scientific practices/governing structures leading to limitations in ILK integration and IP leadership over resource management. | Policy responses: recognize Indigenous usufruct rights, and support the sovereignty of Indigenous Peoples by advocating for local management and control over project lands  Future research: NbS projects need a more inclusive and expansive framework that selects and appropriately engages the right stakeholders. “Nature-based thinking" (e.g., focused on the intrinsic value of nature, to help bridge 'divide' between scientific community and IPLCs) should be adopted where community-centred discourse elevates inherent values of nature, since communities that regularly interact with ecosystems know best how to adapt and maximize beneficial services. NbS projects should be developed in a way that is respectful/inclusive/ accommodating of IPLCs and support the sovereignty of Indigenous Peoples. |
| Zimmermann et al. (2023). A leverage points perspective on Arctic Indigenous food systems research: a systematic review. Sustainability Science, 1-20. | Three important gaps in the current knowledge base are identified that may hinder transformative change: 1) most research conducted about rather than within Arctic Indigenous food systems, there therefore a risk of creating a research environment where predominantly Western scientific concepts are applied to Arctic Indigenous food systems that may be inapplicable to local contexts; 2) past scientific studies have tended to focus on components of Arctic Indigenous food systems in isolation, thereby neglecting to address systemic root causes of complex sustainability challenges and the relevance of interlinkages across system levels; 3) while the number of multi-, inter-, and transdisciplinary research projects has increased over the last decades, most research has to date been disciplinary which can lead to complex inter-relationships not being recognized. |  | Future research: The authors propose inquiries to reflect on traditional scientific approaches and actively contribute to the decolonization of research practices. They further emphasize the significance of interlinkages between shallow and deep leverage points as essential to meet increasingly complex sustainability challenges and encourage inter- and transdisciplinary research collaborations in action-oriented settings. A final suggestion is to co-produced complexity approaches as promising to leverage just and sustainable transitions within Arctic Indigenous food systems. |
| Lebel et al. (2022). Climate change and Indigenous mental health in the Circumpolar North: A systematic review to inform clinical practice. Transcultural Psychiatry, 59(3), 312-336. | Climate change is leading to profound impacts such as cultural disruption, increased stress and anxiety. It affects Indigenous mental health by constraining access to the land and altering the land's resources. Further, impacts on mental health are felt through disruptions of knowledge systems and cultural identities.  Relationships with the land play important role in differentiating climate impacts on mental health - individuals with identities/social roles more closely tied to the environment were more impacted by the changes.  Determinants of adaptation to climate change included higher income, sex and age (e.g., young men's hunting tied to self-worth; Elders worried for potential impacts on future generations) | Authors note how climate change exacerbates socio-economic conditions, which make IP more vulnerable to climate impacts  Environmental changes may impact mental health services by increasing demand, preventing health workers from accessing land in leisure time, increasing communication outages during extreme weather, and compromising land-based mental health programs. Further, climate change is impacting infrastructure that could limit access to health care more widely and overall negatively affect community and individual health.  Youth expressed fear about life-changing circumstances and concerns about how community members were using negative ways of coping. Elders expressed worry for potential impacts on identities, livelihoods of peoples and future generations. | Community or population level: - Indigenous led initiatives can reinforce adaptation/wellbeing through awareness (e.g. , monitor climate change through Indigenous lens considered as way to reinforce adaptation to environmental changes, where community members feel their awareness and response to these changes empowered them to give back to the land, in turn reinforcing cultural connection/attachment to land), cultural connection (strong social capital and cultural integrity are linked to better adaptation to environmental changes), and empowerment (sharing information about climate change benefits through increased awareness, facilitating adaptation of TK, support land safety, and enables people to advocate for own health).   Policy responses (specific to clinicians): - clinicians should be sensitive to health inequities stemming from climate change, and also be attentive to asymmetries in power within the clinic. - clinicians should be aware of and inquire about potential effects of environmental changes on patients, their families, and their communities, to better position effort to assist in adaptation. |
| Shafiee et al. (2022). Food Security Status of Indigenous Peoples in Canada According to the 4 Pillars of Food Security: A Scoping Review. Advances in Nutrition, 13(6), 2537-2558. | Evidence from across Canada indicates that all dimensions of food security are currently being affected among Indigenous Peoples. Climate change has affected all 4 pillars of food security among Indigenous Peoples over time.  The observed decrease in plant and animal health in the Arctic regions has led Indigenous peoples to become more selective about the quality of country foods for consumption  Several cultural and ecological factors have contributed to reduced engagement in traditional subsistence activities, including the poor transfer of cultural knowledge to youth, reduced plant and animal species, climate change, environmental contamination, time and energy required for harvesting, land use, and harvesting practices  Climate change has affected all 4 pillars of food security among Indigenous peoples over time | Climate change has affected all four pillars of food security through direct and indirect mechanisms and processes; 27 references mentioning the topic. | Broadly: The findings of this study suggest that solving food insecurity issues among Indigenous Peoples in Canada, especially those living in remote communities, requires an integrated approach encompassing culturally specific interventions targeting food availability, food cost, food knowledge, food safety, and food quality.  Policy-level: Addressing food insecurity in Indigenous people requires promoting policies that decolonize food and knowledge systems, empower Indigenous people, reconnect Indigenous people with cultural practices, and make food sovereignty possible. |
| Hagen et al. (2022). Climate change-related risks and adaptation potential in Central and South America during the 21st century. Environmental Research Letters, 17(3), 033002. | pg.5 (Table 1) - see for added detail on adaptation options for each risk, as per the reviewed literature (also Table A1 at end of paper)  Cascading/compounding risks (much more significant than individual; overwhelm infrastructure and public service systems); depends on severity of climate hazards; "tipping points of socio-economic systems" and "vulnerability and resilience of these systems, e.g., health, sanitation, energy systems" (p.16)  There are also knowledge gaps for region's capacity to address climate change-related risks. | Record does attend to the socio-economic disparities experienced by IPs in Latin America which enhance vulnerability to climate change effects  Important context (pg. 4): "currently about 800 Indigenous groups in Latin America (incl. Mexico), making up 8% of the population; "Indigenous communities often lack access to infrastructure and public service systems, as well as territorial autonomy and self-determination, and are often forced to occupy climate risk prone areas such as low-lying coastlines, steep slopes, and floodplains"; "disproportionate vulnerability to climate change", due to "widespread inequalities and discrimination". Many groups are "excluded from or have limited influence in political decisions surrounding climate change" (pg.6)   Record also notes that research suggests all levels of decision-making about land and forest should include Indigenous knowledge systems to improve fire management, soil health, ecosystem restoration. Notes that although these systems are disappearing/undermined, they are emerging as mechanisms for climate adaptation | Community-level: choose different crops depending on season/year to accommodate drought periods.  Regional-level: need more reliable climate and environment forecast/monitoring and warning systems for drought/flood risk  Policy responses: clear communication on needs and preferences between local government and residents to avoid misunderstandings/tensions in cases of relocation from high-risk areas. Futher, water resource management pursued in participatory approaches with Indigenous and local knowledge, as well as efficient water storage and diversification of water sources. Investments in distributed and well-equipped public health facilities & surveillance systems to monitor vector development and disease outbreaks. Efforts should be made to reduce deforestation/increase reforestation, and create marine protected areas through better access/integration of scientific knowledge in decision-making processes and management plans  Future research: increased investment on in-depth studies to climate change and socioeconomic development and updated management policies/measures to reduce risks |
| Leal Filho et al. (2022). Understanding responses to climate-related water scarcity in Africa. Science of the Total Environment, 806, 150420. | Most (90%) responses identified lack consistent coordination for implementation at scale, which reveals inconsistent engagement and acknowledgement of local and Indigenous knowledge and practices leading to inconsistent integration of these with formal risk reduction/adaptation strategies | Record mentions non-climatic drivers that make people more vulnerable to water scarcity (and climate change) such as age, income, geography (Rurality), and gender.  Most studies (71%) reported limited scope for adaptation responses, confined to local areas or local communities - and actions with limited scope have common relationship with use of local and Indigenous knowledge. Medium scope actions indicate ability to scale is contingent on role of State and degree of local actor engagement; while 3% articles indicated broad scope actions which resulted in system-wide changed involving entire organization, country, or large region/population  Advancing climate change literacy rates and extension of cli-mate information services provides two low-hanging opportunities that will enhance platforms for adaptation at future warming levels. | Community or population level: In Ethiopia's Tigray Region, Indigenous water strategies (percolation pits/ponds, check-dams, deep trenched bunds) have successfully restored landscape restoration indicating potential benefit for larger scale environmental outcomes. Responses informed by local and Indigenous knowledge include early warning systems predicting seasons, rainwater harvesting practices, stockpiling grains, conservation farming practices and traditional food preservation, but little is known about the role IK plays in urban responses nor the adaptation limits of such practices. Finally, climate change literacy varies across continent, average rate of 37%, which may limit informal and autonomous responses  Regional level: need to extend planned adapation at the local level and better integrate project climate risks into local autonomous responses (including Indigenous responses). Advancing climate literacy rates and climate information services will enhance platforms for future adaptation at future warming levels.  Future research: future work should focus on the evidence of limitations of policy and top-down decision making in deploying responses to water scarcity in Africa and establishing adaptation limits of responses to water scarcity. |
