# Supplementary material for: Climate change, biodiversity loss, and Indigenous Peoples’ health and wellbeing: A systematic umbrella review
Source: PLOS Glob Public Health. 2024 Mar 20;4(3):e0002995. doi: 10.1371/journal.pgph.0002995 (PMC10954122; doi:10.1371/journal.pgph.0002995)
Supplement: S3 Table — (DOCX) [file pgph.0002995.s004.docx]

**S3 Table. Grey literature records retrieved and included in the review.**

| **Document Type** | **Reference** |
| --- | --- |
| **Global** | |
| Meeting Report | Special Rapporteur on Rights of Indigenous Peoples, A/HRC/51/28. In United Nations General Assembly, Human Rights Council: Fifty First Session, 12 Sept-7 Oct 2022 ,. [Indigenous women and the development, application, preservation and transmission of scientific and technical knowledge Report of the Special Rapporteur on the rights of indigenous peoples](https://www.ohchr.org/en/documents/thematic-reports/ahrc5128-indigenous-women-and-development-application-preservation-and); 2022. |
| Report | IPBES (2019): [Global assessment report on biodiversity and ecosystem services of the Intergovernmental Science-Policy Platform on Biodiversity and Ecosystem Services](https://www.ipbes.net/global-assessment). E. S. Brondizio, J. Settele, S. Díaz, and H. T. Ngo (editors). IPBES secretariat, Bonn, Germany. |
| Report | IPBES (2018): T[he IPBES assessment report on land degradation and restoration](https://www.ipbes.net/assessment-reports/ldr). Montanarella, L., Scholes, R., and Brainich, A. (eds.). Secretariat of the Intergovernmental Science-Policy Platform on Biodiversity and Ecosystem Services, Bonn, Germany. |
| Report | IPBES (2022). [Methodological Assessment Report on the Diverse Values and Valuation of Nature of the Intergovernmental Science-Policy Platform on Biodiversity and Ecosystem Services](https://zenodo.org/record/7687931#.ZC7YcHZByUk). Balvanera, P., Pascual, U., Christie, M., Baptiste, B., and González-Jiménez, D. (eds.). IPBES secretariat, Bonn, Germany |
| Report | ILO (2022). [Indigenous Peoples and Climate Change: Emerging Research on Traditional Knowledge and Livelihoods.](https://www.ilo.org/wcmsp5/groups/public/---ed_protect/---protrav/---ilo_aids/documents/publication/wcms_686780.pdf) Ahearn, A., Oelz, M., Kumar Dhir, R. (eds). International Labour Office, Geneva, Switzerland. |
| Meeting Report | E/C.19/2023/5. In United Nations Economic and Social Council, Permanent Forum Indigenous Issues: Twenty-Second Session, 17-28 April 2023. [Indigenous determinants of health in the 2030 Agenda for Sustainable Development](https://documents-dds-ny.un.org/doc/UNDOC/GEN/N23/029/12/PDF/N2302912.pdf?OpenElement); 2023. |
| Report | FAO and Alliance of Biodiversity International and CIAT (2021). [Indigenous Peoples’ food systems: Insights on sustainability and resilience in the front line of climate change](https://www.fao.org/3/cb5131en/cb5131en.pdf). Rome |
| Report | Van Uffelen, A., Tanganelli, E., Gerke, A., Bottigliero, F., Drieux, E., Fernández-de-Larrinoa, Y, et al. (2021). [Indigenous youth as agents of change – Actions of Indigenous youth in local food systems during times of adversity](https://www.fao.org/documents/card/en/c/cb6895en/). Rome, FAO. |
| Report | IPCC (2019): Technical Summary  In: [IPCC Special Report on the](https://www.ipcc.ch/site/assets/uploads/sites/3/2022/03/02_SROCC_TS_FINAL.pdf)  [Ocean and Cryosphere in a Changing Climate](https://www.ipcc.ch/site/assets/uploads/sites/3/2022/03/02_SROCC_TS_FINAL.pdf) [Pörtner, HO., Roberts, D.C., Masson-Delmotte, V., Zhai, P., Poloczanska, E., Mintenbeck, K. et al. (eds.)]. Cambridge University Press, Cambridge, UK and New York, USA |
| Report | IPCC (2022): Climate Change 2022: [Impacts, Adaptation and Vulnerability. Contribution of Working Group II to the Sixth Assessment Report of the Intergovernmental Panel on Climate Change](https://report.ipcc.ch/ar6/wg2/IPCC_AR6_WGII_FullReport.pdf) [Pörtner, HO., Roberts, D.C., Tignor, M.,. Poloczanska, E.S., Mintenbeck, K., Alegría, A.M. et al. (eds.)]. Cambridge University Press. Cambridge University Press, Cambridge, UK and New York, USA |
| **Africa** | |
| Policy Brief | Eronmhonsele, J., Adejeghwro, M.O. (2019): [CPD Policy Brief: Climate Change and its implication on Women’s Health in the Niger Delta Region](https://africaportal.org/wp-content/uploads/2023/05/Climate_change_and_its_implication_on_womens_health-1.pdf). Centre for Population and Envrionmental Development, Benin City, Nigeria. |
| Policy Brief | UNESCO. (2018). [Indigenous and local knowledge, biodiversity and climate change](https://unesdoc.unesco.org/ark:/48223/pf0000366831). LiNKS. |
| Report | World Meteorological Organization (2022). [State of the Climate in Africa: 2021](https://library.wmo.int/doc_num.php?explnum_id=11512). Geneva, Switzerland. |
| Report | Sutz, P, Beauchamp, E and Bolin, A (2021) [Routes to change: rural women’s voices in land, climate and market](https://www.iied.org/sites/default/files/pdfs/2021-08/20331iied.pdf)  [governance in sub-Saharan Africa Research report.](https://www.iied.org/sites/default/files/pdfs/2021-08/20331iied.pdf) IIED, London. |
| Report | World Food Programme (2021). [Climate Change in Southern Africa](https://executiveboard.wfp.org/document_download/WFP-0000129015). Regional Bureau Johannesburg, South Africa. |
| Report | Miller, K.A., Mcunu, N., Anhauser, A., Farrow, A., Santillo, D., Johnston, P. (2020). [Weathering the Storm: Extreme Weather events and climate change in Africa](https://www.greenpeace.org/static/planet4-africa-stateless/2020/11/b6e9a1fa-weathering-the-storm-extreme-weather-events-and-climate-change-in-africa-grl-trr-04-2020-high-res.pdf). GreanPeace Africa. |
| Report | UNICEF (2022). [Regional Call to Action: Horn of Africa Drought Crisis: Climate Change is here now.](https://reliefweb.int/report/somalia/regional-call-action-horn-africa-drought-crisis-climate-change-here-now-may-2022) UNICEF Eastern and Southern Africa Regional Office, Nairobi, Kenya. |
| Website Post | Sanago, G. (2022). [How Indigenous Peoples in Africa are impacted by climate change.](https://www.iwgia.org/en/news/4959-how-indigenous-peoples-in-africa-are-impacted-by-climate-change.html) Indigenous Debates. |
| Presentation | UNESCO (2021). [Knowing our changing climate in Africa](https://en.unesco.org/sites/default/files/links_knowingclimateafrica_2021.pdf). LiNKS |
| Meeting Report | UNESCO Office Nairobi and Regional Bureau for Science in Africa. [Report of the UNESCO Expert Meeting on indigenous Knowledge and Climate Change in Africa](https://unesdoc.unesco.org/ark:/48223/pf0000374999.locale=en), Nairobi, Jenya, 27-28, June 2018. |
| Report | IWGIA (2021). [Addressing Indigenous People’s challenges through capacity enhancement and advocacy on change](https://pingosforum.or.tz/wp-content/uploads/2022/11/Climate-Change-Report-2021.pdf). Pingo’s Forum. |
| **Latin America & Caribbean** | |
| Report | Minority Rights Group International (2019). Chapter 11: Peru: Hunger and Malnutrition among Shawi communities in the Amazon. [Minority and Indigenous Trends: Focus on Climate Justice.](https://minorityrights.org/wp-content/uploads/2020/08/2019_MR_Report_170x240_V7_WEB.pdf) London, UK. |
| Book | Kronik, J. & Verner, D. (2013). Indigenous Peoples and Climate Change in Latin America and the Caribbean. Directions in Development- Environment and Sustainable Development. [DOI: 10.1596/978-0-8213-8237-0](https://elibrary.worldbank.org/doi/abs/10.1596/978-0-8213-8237-0) |
| Workshop Report | SC-SII/2020/ME/ILKCC/CBN. UNESCO (2020). [Workshop report: Mobilizing Indigenous and Local Knowledge Solutions: Addressing Climate Impacts and Vulnerabilities, a Perspective from the Caribbean Region](https://unesdoc.unesco.org/ark:/48223/pf0000375025?posInSet=2&queryId=N-EXPLORE-7bf31ac0-49ef-45a7-85cb-6a1e17a669ae), Georgetown, Guyana, 3-5 September 2019 |
| Report | FAO (2021). Indigenous peoples, Afro-descendants and climate change in Latin America: [Ten scalable experiences of intercultural collaboration.](https://www.fao.org/3/cb4847en/cb4847en.pdf) Santiago. |
| Report | The Nature Conservancy (2021). [Latin America Impact Report.](https://www.nature.org/en-us/about-us/where-we-work/latin-america/) |
| Report | IWGIA (2020). Annual Report 2020; Annual Report. |
| Report | World Meteorological Organization (2021). [State of the Climate in Latin America and the Caribbean](https://reliefweb.int/report/world/state-climate-latin-america-and-caribbean-2021). Analysis. |
| **Australia** | |
| Policy Report | Indigenous Peoples’ Organisation-Australia (2021). [Heal Country, Heal Climate:](https://www.ohchr.org/sites/default/files/2022-03/indigenous-peoples-organization2.pdf) Priorities for climate and environment. |
| Discussion Paper | Lowitja Insitute & National Health Leadership Forum (2021). [Climate Change and Aboriginal and Torres Strait Islander Health.](https://www.lowitja.org.au/content/Image/Lowitja_ClimateChangeHealth_1021_D10.pdf) Healthy Environments and Lives (HEAL) Network, Centre for Excellence in Strengthening Systems for Indigenous Health Care Equity. |
| Discussion Paper | National Aboriginal Community Controlled Health Organization (2019). [Mitigating Climate Change to Optimize Aboriginal and Torres Strait Islander Health](https://www.health.wa.gov.au/~/Media/Files/Corporate/General%20documents/Climate%20health%20wa%20inquiry/Public%20submissions/Organisations/Nationalaboriginalcommunitycontrolledhealthorganisation.Pdf) |
| **New Zealand** | |
| Research Report | Manaaki Whenua Landcare Research (2021). [He Huringa ahuarangi, he huringa ao: a changing climate, a changing world.](https://www.landcareresearch.co.nz/assets/researchpubs/He-huringa-ahuarangi-he-huringa-ao-a-changing-climate-a-changing-world.pdf) Nga Pae o te Maramatanga. |
| Policy Report | Ministry for the Environment (2020). [Ngā mahi āhuarangi mō ngāi Māori Climate action for Māori.](https://environment.govt.nz/assets/publications/NAP-info-sheets-August-2022/Climate-action-for-Maori-The-national-adaptation-plan-v2.pdf) Aotearoa New Zealand first national adaptation plan. |
| **Asia** | |
| Policy Report | UNDP (2022). Nationally Determined Contributions in Asia: Are Governments Recognizing the Rights, Roles and Contributions of Indigenous Peoples? Asia Indigenous Peoples Pact Foundation. |
| Policy Brief | UN Human Rights South-East Asia Regional Office (2022). Environmental Rights Brief: Climate Justice in Southeast Asia. UN Environment Programme. |
| Policy Brief | International Network of Mountain Indigenous Peoples (2018). [The Suusamyr Declaration](https://www.iied.org/g04342) |
| **Oceania** | |
| Research Brief | POCCA Scientific Steering Committee (2022). [Pacific Ocean Climate Crisis Assessment](https://unfccc.int/sites/default/files/resource/202209201059---POCCA%20Stocktake%20Submission_11-Aug-2022.pdf). Submission to the UNFCC Global Stocktake. |
