# Supplementary material for: Climate change, biodiversity loss, and Indigenous Peoples’ health and wellbeing: A systematic umbrella review
Source: PLOS Glob Public Health. 2024 Mar 20;4(3):e0002995. doi: 10.1371/journal.pgph.0002995 (PMC10954122; doi:10.1371/journal.pgph.0002995)
Supplement: Attachment — Submitted filename: Brubacher PLOS Global Public Health comments peer review.docx [file pgph.0002995.s005.docx]

PGPH-D-23-02151
Climate change, biodiversity loss, and Indigenous Peoples' health and wellbeing: A systematic umbrella review

**Peer reviewer’s comments:**

Thank you for your interest in supporting the health and wellbeing of Indigenous Peoples. It is a pleasure to review an article that is so close to being a final draft ready for publication. The consistently clear writing and application of sufficient and relevant references is laudable. As follows are a few comments and some minor suggestions for improvement. Suggested changes are highlighted.

1. Good methodology (umbrella review) chosen
2. Line 100 suggestion to revise “…biodiversity loss impacts on Indigenous Peoples’ health….
3. Line 104 “…need for a comprehensive literature review in conjunction with forthcoming work on climate change…”

Alternatively, could be “need for a knowledge synthesis review to support forthcoming work on…”

4. Line 131 “(February 2023 meeting) and provided critical feedback….”
5. Line 136 “The sensitivity of the search approach was enhanced by a manual search of the following journals:”
6. Paragraph Lines 163-173: While the whole paper is clearly written I found that this paragraph about the technique of using the NVivo software was somewhat muddied. It lacks detail and clarity. A little more information or even a statement on where to find some examples or instructions would be helpful. Perhaps it is just me. I have been interested in using such a technique for a while and your imprecise description both whetted my appetite and left me confused as to how this really worked for your synthesis.
7. Line 521 “…or a broader steering committee [36,60], or demonstrated that Indigenous peoples had input on the final manuscript to ensure…”
8. Lines 531-547 – very well written. It seems like this section would be a good place to include a specific reference or statement about UNDRIP.

There was one drawback to my ability to provide constructive peer review. That was the reality that the protocol has not been published. In such cases, it would be helpful for the editor to include the draft submission of the separately published protocol with the analysis article.

9. Line 569 “To promote health equity, increase collaboration, and shift power differentials, this evolving determinant of health discourse has bridged social justice movements in unique ways.” *This statement is clunky and should be rewritten to clearly emphasize what you want to state.*
10. You might want to add another limitation (section begins on line 616) Since it is based on a review of reviews/umbrella review, there is always the limitation that the information is not quite as current or fresh as if it was a standard systematic or scoping review of studies. That lag time does clearly impact on anything related to climate change since so much is being published on this right now. Most reviews you include would be using studies that were published at least a year or two ago, and likely 5 to 8 years ago.

Comments on references (if I am wrong, the editor will know best)

1. Great selection of references. High praise for being thorough and using a good balance of peer reviewed literature, grey literature, reports and government or NGO publications.
2. The abbreviations for some of the journals seem to be incorrect. E.g. *Wellbeing, Sp Soc* should be Wellbeing Space Soc
3. *PLOS Glob Public Heal* should be PLOS Glob Public Health
4. *Lancet Planet Heal* should be Lancet Planet Health
5. *One Heal* should be One Health

You should be able to look up the correct journal abbreviations in PubMed, Journals database <https://www.ncbi.nlm.nih.gov/nlmcatalog/journals/>
