# Supplementary material for: Climate change, biodiversity loss, and Indigenous Peoples’ health and wellbeing: A systematic umbrella review
Source: PLOS Glob Public Health. 2024 Mar 20;4(3):e0002995. doi: 10.1371/journal.pgph.0002995 (PMC10954122; doi:10.1371/journal.pgph.0002995)
Supplement: Attachment — Submitted filename: Response to Reviewers.docx [file pgph.0002995.s006.docx]

**Manuscript ID: PGPH-D-23-02151:** *"Climate Change, Biodiversity Loss, and Indigenous Peoples' Health and Wellbeing: A Systematic Umbrella Review"*

**Reviewer #1:**

**This is a brilliant review and synthesis of the available literature, with sound methodology and an appropriate and ethnical approach (i.e. Indigenous consultation and oversight). The breadth and detail of the findings are laudable, with a sound discussion that pulls together the threads and provides a clear path forward for future research. As a very minor suggestion - because settler colonial studies and elements of critical Indigenous studies have been at pains to distinguish settler colonialism from other forms (i.e. franchise or exploitation colonialism), and because there's a growing literature and focus on settler colonialism and health - it may be worth at least noting in summary statements/the discussion that you are including settler colonialism within the umbrella of colonialism. And of course if any of the papers specified or distinguished, this might be helpful to tease out as well. Thank you for this excellent, high-quality, and very useful addition to the literature.**

Author's Response: Thank you for your thoughtful comments and affirmation. We appreciate the time you have given to reviewing our manuscript.

Thank you for your insightful comment regarding distinguishing settler colonialism from other forms. We have made more explicit in the text that we are including settler colonialism within the umbrella of colonialism, as follows:

- Pg.27, Line 600: “*Colonialism, including settler colonialism, is increasingly recognized as a determinant and driver of climate-health and biodiversity impacts [8,12,83,84]”*

Additionally, we have added a sentence to the results to reference the few articles that named settler colonialism, specifically, within the article. As indicated below, the majority of reviews used the umbrella term and did not distinguish between settler colonialism and other forms:

- Pg.24, Lines 507-509: *“Three reviews (7.9%) named settler colonialism, specifically, in the framing of the review [26,42] and situating of results [38], whereas most of the reviews discussing colonialism used the term broadly (i.e., to possibly include other forms such as franchise or exploitation colonialism).”*

**Reviewer #2: All comments attached are for authors and editors. This submission needs little revision to be ready for publication. As far as I could tell, the availability of data will be met. This is a quality journal article submission.**

**Thank you for your interest in supporting the health and wellbeing of Indigenous Peoples. It is a pleasure to review an article that is so close to being a final draft ready for publication. The consistently clear writing and application of sufficient and relevant references is laudable. As follows are a few comments and some minor suggestions for improvement. Suggested changes are highlighted.**

Author's Response: Thank you for your detailed and thoughtful feedback on our manuscript. We have responded point-by-point to each of your comments below.

**1. Good methodology (umbrella review) chosen**

Author’s Response: Thank you for this comment.

**2. Line 100 suggestion to revise “…biodiversity loss impacts on Indigenous Peoples’ health….**

Author’s Response: Thank you for this suggestion. We have revised Line 102 accordingly, and have also replaced other instances of “impacts to” with “impacts on” throughout the manuscript (lines 26, 84, 369, 433, 599).

**3. Line 104 “…need for a comprehensive literature review in conjunction with forthcoming work on climate change…” Alternatively, could be “need for a knowledge synthesis review to support forthcoming work on…”**

Author’s Response: Thank you for these suggestions. We have revised this sentence accordingly (pg.4, Lines 106-107): *“*...*identified the need for a comprehensive literature review to support forthcoming work on climate change, biodiversity, and health”.*

**4. Line 131 “(February 2023 meeting) and provided critical feedback….”**

Author’s Response: Thank you for this suggestion. We have revised this sentence accordingly (now pg.5, Line 135).

**5. Line 136 “The sensitivity of the search approach was enhanced by a manual search of the following journals:”**

Author’s Response: Thank you for this suggestion. We have revised this sentence accordingly (now pg.5, Line 140).

**6. Paragraph Lines 163-173: While the whole paper is clearly written I found that this paragraph about the technique of using the NVivo software was somewhat muddied. It lacks detail and clarity. A little more information or even a statement on where to find some examples or instructions would be helpful. Perhaps it is just me. I have been interested in using such a technique for a while and your imprecise description both whetted my appetite and left me confused as to how this really worked for your synthesis.**

Author’s Response: Thank you for this comment. We have revised this section to add detail on how we used *NVivo* software, and particularly how we used case attributes and classifications. Given the multidisciplinary and methodologically diverse readership of *PLOS Global Public Health*, however, we have intentionally limited the extent of technical detail provided. The use of case attributes and classifications and the querying function within *NVivo* software can quickly become complicated and require very granular detail to explain. As such, we have focused on adding information that might be helpful for a reader to know in order to understand the results of our study, rather than to grasp the technical functions of the software.

The new text reads as follows (italicized and underlined), pgs.6-7, Lines 178-186: *“Building from these insights, two team members (LJB, LP) conducted detailed line-by-line coding of the extraction and quality appraisal frameworks, using* NVivo Release 1.5 *software for organization and retrieval of codes and coded excerpts. Case attributes and classifications corresponding to data extraction and quality appraisal domains were applied to articles within* NVivo *to enable querying of co-occurrence of codes across reviews and to identify potential trends in the data (e.g., co-occurrence of a given ‘geographic location’ attribute with a high level of Indigenous Peoples’ involvement in the research; or a ‘study discipline’ attribute with the type of climate responses recommended by articles).”*

**7. Line 521 “…or a broader steering committee [36,60], or demonstrated that Indigenous peoples had input on the final manuscript to ensure…”**

Author’s Response: Thank you for this suggestion. We have revised this sentence to now read (pg.25, Lines 550-553): *“Review methodologies were informed by Indigenous partners or a broader steering committee [36,60], or reviews reported that Indigenous Peoples had input on the final manuscript to ensure framing aligned with their priorities [30].”*

This revised phrase more accurately reflects what we are able to claim about the reviewed literature. That is, we can only ascertain the level of input of Indigenous Peoples in a given study from what a reviewed article reported explicitly versus demonstrated in some way.

**8. Lines 531-547 – very well written. It seems like this section would be a good place to include a specific reference or statement about UNDRIP.**

Author’s Response: Thank you for this suggestion. We agree that this paragraph is a very appropriate place to connect our results to the UNDRIP. We have added a reference to the UNDRIP at the end of the paragraph, as follows (pg.26, Line 579):

*“Responses to climate change and biodiversity loss, then, need to interrogate the locus of power – question who is holding it – and move towards a model of equitable and shared decision-making [70], focused on supporting Indigenous Peoples’ sovereignty, rights, and autonomy as outlined in the UNDRIP.”*

**9. There was one drawback to my ability to provide constructive peer review. That was the reality that the protocol has not been published. In such cases, it would be helpful for the editor to include the draft submission of the separately published protocol with the analysis article.**

Author’s Response: Thank you for this comment. Since submission of this manuscript, our protocol manuscript has been published in *BMC Systematic Reviews*. The corresponding reference to the protocol in the text has been revised with the updated citation information, and can be found here:

*Brubacher LJ, Chen TT-W, Longboat S, Dodd W, Peach L, Elliott SJ, et al. Climate change and Indigenous Peoples’ health and wellbeing: A systematic umbrella review protocol. Syst Rev. 2024;13(8):1-9. doi: 10.1186/s13643-023-02423-x*

**10. Line 569 “To promote health equity, increase collaboration, and shift power differentials, this evolving determinant of health discourse has bridged social justice movements in unique ways.” *This statement is clunky and should be rewritten to clearly emphasize what you want to state.***

Author’s Response: Thank you for this comment. In re-reading this paragraph, we realized the first few sentences were unclear and some statements unnecessary, including the sentence you highlighted. For clarity and concision, we have revised this section to now read as follows:

Pg.27, Lines 600-606: *“Colonialism, including settler colonialism, is increasingly recognized as a determinant and driver of climate-health and biodiversity impacts [8,12,83,84]. ~~To promote health equity, increase collaboration, and shift power differentials, this evolving determinant of health discourse has bridged social justice movements in unique ways [85]. Although these health movements are of great importance~~ This recognition is important, yet Indigenous scholars and advocates have indicated that ~~there is something missing~~ it is unclear where planetary health fits into the existing determinants of health language [8]. ~~It has been proposed that~~ An ecocentric approach is needed, a critical repositioning of human engagement with the land and environments, in our understanding of determinants of health and wellbeing [75,83,86] and to better inform research, policy, and community-led responses.*

**11. You might want to add another limitation (section begins on line 616) Since it is based on a review of reviews/umbrella review, there is always the limitation that the information is not quite as current or fresh as if it was a standard systematic or scoping review of studies. That lag time does clearly impact on anything related to climate change since so much is being published on this right now. Most reviews you include would be using studies that were published at least a year or two ago, and likely 5 to 8 years ago.**

Author’s Response: Thank you for this suggestion. In response, we have added the following statement to the limitations section (new text is underlined):

Pg.29, Lines 657-665: *“Importantly, the umbrella review methodology relies on already-synthesized insights across the published literature. This may have limited the depth and breadth of findings related to climate-health and biodiversity impacts, particularly if the secondary literature is not as geographically or topically diverse as the primary. We also may not have captured the most current published literature through this review of reviews. We aimed to address these limitations through a robust search and integration of the unpublished and current 'grey' literature, focused particularly on geographic gaps in published reviews, as well as our application of a weighted criteria to include additional reviews from geographic regions or populations less represented in the published literature.*

**12. Comments on references (if I am wrong, the editor will know best)**

- **Great selection of references. High praise for being thorough and using a good balance of peer reviewed literature, grey literature, reports and government or NGO publications.**
- **The abbreviations for some of the journals seem to be incorrect. E.g. *Wellbeing, Sp Soc* should be Wellbeing Space Soc**
- ***PLOS Glob Public Heal* should be PLOS Glob Public Health**
- ***Lancet Planet Heal* should be Lancet Planet Health**
- ***One Heal* should be One Health**

**You should be able to look up the correct journal abbreviations in PubMed, Journals database** [**https://www.ncbi.nlm.nih.gov/nlmcatalog/journals/**](https://www.ncbi.nlm.nih.gov/nlmcatalog/journals/)

Author’s Response: Thank you for flagging these errors. We have carefully reviewed the accuracy of the referencing and have revised accordingly, including making the changes to journal abbreviations that you noted above.

Thank you again to both of the reviewers for their thorough and constructive review of our manuscript.
